# Economic burden of heart failure in Europe: A systematic review of costs and cost‐effectiveness

**DOI:** 10.1002/ehf2.70017

**Published:** 2025-11-26

**Authors:** Josep Darbà, Meritxell Ascanio, Antonio Rodríguez, Sarah J. Charman, Nduka C. Okwose, Renae J. Stefanetti, Amy Groenewegen, Annamaria Del Franco, Maria Tafelmeier, Andrej Preveden, Amy S. Fuller, Fatima Bano, David R. Sinclair, Duncan Edwards, Anne P. Nelissen, Petros N. Malitas, Aikaterini Zisaki, Zoran Bosnić, Petar Vračar, Alessandra Fornaro, Fausto Barlocco, Dimitris Fotiadis, Prithwish Banerjee, Guy A. MacGowan, Oscar Fernandez, José Luis Zamorano, Marta Jimenez‐Blanco Bravo, Lars S. Maier, Iacopo Olivotto, Massimo Milli, Frans H. Rutten, Jonathan Mant, Lazar Velicki, Petar M. Seferovic, Nenad Filipovic, Djordje G. Jakovljevic, STRATIFYHF investigators, Laura Sasso, Alessandra Milazzo, Giulia Taborchi, Serena Fatucchi, Aleksandra Milovancev, Kate Williams, Patience Jecheche, Borut Flis, Dimitrios Boucharas, Dimitris Manousos, Manolis Tsiknakis, Thomas Kassiotis, Tijana Sustersic, Bogdan Milicevic, Marija Gacic, Milica Kaplarevic, F.D. Richard Hobbs, Onno Kaagman

**Affiliations:** ^1^ Department of Economics Universitat de Barcelona Barcelona Spain; ^2^ BCN Health Economics and Outcomes Research S. L, Travessera de Gràcia Barcelona Spain; ^3^ Translational and Clinical Research Institute, Faculty of Medical Sciences Newcastle University Newcastle upon Tyne UK; ^4^ Newcastle upon Tyne Hospitals NHS Foundation Trust Newcastle upon Tyne UK; ^5^ Clinical Sciences and Translational Medicine Research Theme, Research Centre for Health and Life Sciences, Institute of Health and Wellbeing Coventry University Coventry UK; ^6^ University Hospitals Coventry and Warwickshire NHS Trust Coventry UK; ^7^ Wellcome Centre for Mitochondrial Research. Translational and Clinical Research Institute, Faculty of Medical Sciences Newcastle University Newcastle upon Tyne UK; ^8^ National Institute for Health and Care Research (NIHR) Newcastle Biomedical Research Centre (BRC) Newcastle upon Tyne UK; ^9^ Department of General Practice & Nursing Science, Julius Centre for Health Sciences and Primary Care, University Medical Centre Utrecht Utrecht University Utrecht Netherlands; ^10^ Careggi University Hospital Florence Italy; ^11^ Department of Internal Medicine II University Medical Centre Regensburg Regensburg Germany; ^12^ Faculty of Medicine University of Novi Sad Novi Sad Serbia; ^13^ Institute of Cardiovascular Diseases Vojvodina Sremska Kamenica Serbia; ^14^ Population Health Sciences Institute, Faculty of Medical Sciences Newcastle University Newcastle upon Tyne UK; ^15^ Primary Care Unit, Department of Public Health and Primary Care University of Cambridge Cambridge UK; ^16^ PKNM Solutions Sàrl, Chemin Des Vignes, 2, Colombier (VD) CH ‐ 1114 Commune D'Echichens Vaud Switzerland; ^17^ Faculty of Computer and Information Science University of Ljubljana Ljubljana Slovenia; ^18^ Department of Experimental and Clinical Medicine University of Florence Florence Italy; ^19^ Department of Biomedical Research Foundation for Research and Technology Hellas Heraklion Greece; ^20^ Biosciences Institute Newcastle University Newcastle upon Tyne UK; ^21^ Hospital Universitario Ramón y Cajal Madrid Spain; ^22^ Centro de Investigación Biomédica en Red CIBER‐CV Instituto de Salud Carlos III Madrid Spain; ^23^ Azienda ASL Toscana Centro Florence Italy; ^24^ Department of Cardiology, Faculty of Medicine University of Belgrade Belgrade Serbia; ^25^ Serbian Academy of Sciences and Arts Belgrade Serbia; ^26^ Heart Failure Society of Serbia Belgrade Serbia; ^27^ Bioengineering Research and Development Center, BioIRC Kragujevac Serbia; ^28^ Faculty of Engineering University of Kragujevac Kragujevac Serbia; ^29^ European Primary Care Cardiovascular Society Utrecht Netherlands; ^30^ Oxford Primary Care, Radcliffe Primary Care Building, ROQ University of Oxford Oxford UK

**Keywords:** heart failure, healthcare costs, cost effectiveness, economic burden, resource utilization

## Abstract

Heart failure (HF) affects over 64 million individuals worldwide and is a major cause of hospitalization and mortality, particularly among older adults. In Europe, HF imposes a significant and growing economic burden. This systematic review aimed to evaluate the economic impact of HF diagnosis, treatment and management across European healthcare systems. A systematic literature search was conducted using PubMed, Cochrane Library and Econlit databases including the terms ‘heart failure’ AND ‘costs’ OR ‘cost of illness’ OR ‘cost analysis’ OR ‘economic burden’ OR ‘cost effectiveness’ OR ‘primary care’ OR ‘secondary care’. Studies published between January 2000 and January 2024 were included. A total of 49 studies were included: 17 on resource use, 11 on costs, 15 on resource use and costs, 1 on costs and cost‐effectiveness, and 5 on resource use, costs and cost‐effectiveness. Hospitalizations and medication use were the most frequently reported resource parameters. Annual HF‐related costs varied widely across countries, ranging from €613 to €22,647 per patient. Hospitalizations represented the primary cost driver, accounting for 15% to 92% of total HF costs. Cost‐reduction strategies included multidisciplinary care, telemonitoring and pharmacologic interventions. Several disease management programmes reduced hospital admissions and emergency visits. Cost‐effectiveness analyses supported the use of certain HF therapies, with incremental cost‐effectiveness ratios ranging from €1490 to €9406 per QALY gained. F imposes a substantial economic burden in Europe, largely driven by hospitalizations. Cost‐effective interventions such as remote monitoring and integrated care programmes can reduce this burden. Broader adoption of these strategies may improve outcomes and optimize resource allocation across healthcare systems.

## Graphical abstract

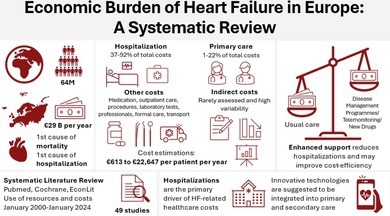

## Introduction

Heart failure (HF) is a widespread epidemic affecting more than 64 million individuals globally.^1^ Diagnosed cases have surged by 12% in the past decade, a rise largely driven by the aging population, underscoring an accelerating trend in HF prevalence worldwide.^1^ People over 55 years of age have a lifetime risk of developing HF, estimated at 33% in men and 28% in women.^2^ HF is the primary cause of hospitalization and death in elderly individuals, resulting in a higher mortality rate compared with many prevalent types of cancer.^3^ Previous research estimated that, within the European Union (EU), HF contributes to 2% of healthcare expenditure, totalling €29 billion annually.^4^ The major cost drivers are hospitalizations, and these expenses are anticipated to increase by 50% over the next 25 years.^5^

The identification, diagnosis and treatment of HF remain challenging, as approximately 74% of patients suspected of having HF present with at least one co‐morbidity, contributing to increased morbidity, mortality and reduced quality of life.^6^ The World Heart Federation considers that the main obstacle in current clinical practice is the limited access to healthcare facilities with qualified experts for risk stratification and early diagnosis of HF.^7^ Consequently, diagnostic inaccuracies can reduce patients' quality of life while increasing resource utilization and the economic burden of HF on healthcare systems.^8^, ^9^ As a result, the overall economic impact of HF in Europe remains unresolved, presenting ongoing challenges in mitigating its strain on healthcare systems.

The objective of this systematic literature review (SLR) was to evaluate the economic impact of current HF practices and the associated costs of HF care in Europe, with a particular focus on assessing their financial burden on healthcare systems. By analysing resource utilization and overall HF‐related expenditures, as well as reviewing studies on cost‐effectiveness, this review sought to quantify the overall economic burden of HF on European healthcare systems.

## Methods

Relevant research was identified through searches in the PubMed, Cochrane Library and Econlit databases. Full‐text articles in English or Spanish published between January 2000 and January 2024 were included in the review.

The selection of the studies was conducted in four phases. The first phase was identification, during which all relevant records were initially gathered, and any duplicate entries were removed. The search terms ‘heart failure’ AND ‘costs’ OR ‘cost of illness’ OR ‘cost analysis’ OR ‘economic burden’ OR ‘cost effectiveness’ OR ‘primary care’ OR ‘secondary care’ were used for the review. In the second phase, screening was completed by assessing the titles and abstracts of the collected papers, with studies not meeting the inclusion criteria excluded at this stage. In the third phase, records that fully met the inclusion criteria and contained the necessary information and variables were selected for inclusion in the analysis (*Table* *1*). Finally, study references were manually screened to include additional articles that were not found in the first search.

**Table 1:** Inclusion criteria applied during the screening

	Inclusion criteria
Disease	HF
Therapy	Any type Primary interest: PC management
Comparator	Any type
Outcome	Use of resources Direct costs, including PC and/or secondary care Indirect costs Total costs Diagnostic procedures
Study design	Articles related to HF costs of inpatient or outpatient care Costs of HF treatment and follow‐up Cost‐of‐illness studies Cost‐effectiveness studies Clinical guidelines for HF diagnosis
Timing	Published between January 2000 and January 2024
Setting	Europe Primary interest: United Kingdom, the Netherlands, Germany, Spain, Italy, Serbia
Languages	English Spanish

HF, heart failure; PC, primary care.

To ensure current relevance, papers published before the year 2000 were not considered due to changes in diagnosis and treatment protocols over time.^10^, ^11^, ^12^, ^13^ Applying inclusion criteria, the review focused on articles related to resource use, costs and cost‐effectiveness. Total costs, costs of primary and secondary care, direct non‐healthcare costs and indirect costs were manually extracted and reported when available. In addition, the information about the cost parameters considered in each assay with their respective perspectives was also extracted. This included the year of cost valuation, duration of follow‐up, currency and discount rates. To enable comparisons between studies, all values were converted to Euros using the exchange rates provided by the Organization for Economic Cooperation and Development (OECD). Conversions were based on the exchange rates from the last year of the costing valuation. Studies that conducted a costing valuation prior to the adoption of the Euro (1 January 1999) were excluded from the cost valuation.

Before screening the articles and abstracts, two reviewers (AR and MA) independently assessed the titles and abstracts based on predefined inclusion and exclusion criteria to ensure the relevance of the selected studies. After this initial evaluation, they collaboratively reviewed the full texts of the chosen articles to determine their eligibility. Any discrepancies between the reviewers were resolved through discussion, leading to a mutually agreed‐upon decision.

## Results

**Figure 1:**
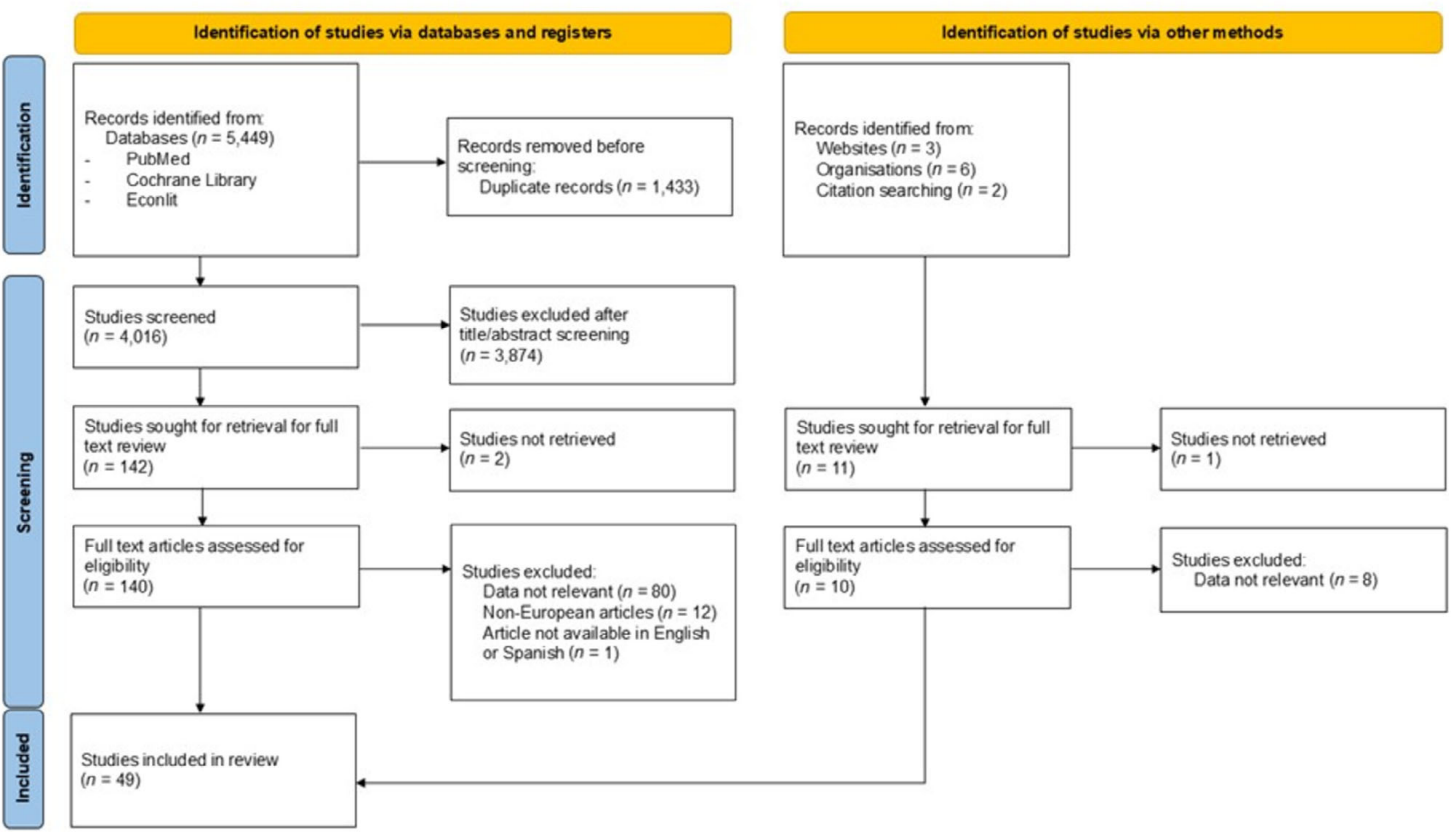
Preferred Reporting Items for Systematic Reviews and Meta‐Analyses (PRISMA) flow diagram of the studies through the review.

A total of 5460 articles were identified. After excluding 1433 records, 4027 title/abstracts were screened. During the screening, 3874 articles were excluded after reviewing the title and the abstract, 3 were not retrieved and 101 were excluded after a full‐text review. Finally, 49 studies met the eligibility criteria for inclusion: 17 regarding HF resource use, 11 regarding HF costs, 15 regarding HF resource use and costs, 1 regarding HF costs and cost‐effectiveness, and 5 regarding HF resource use, costs and cost‐effectiveness (*Table* *2*, *Table* S1 *Figure* *1*).

**Table 2:** General characteristics of studies selected (*n* = 49) (condensed)

Category	Summary of evidence	Key observations/trends
Publication period	2000–2023	Steady increase in publication volume; over 60% of studies published after 2015.
Study design	Cost‐of‐illness, cost‐effectiveness, and mixed economic evaluations	Cost‐of‐illness analyses dominate; cost‐effectiveness studies mainly evaluate pharmacologic or disease‐management interventions.
Data source	Administrative/claims databases, hospital or registry data, and trial‐based analyses	Registry and claims data are most frequent; trial‐based studies provide detailed costing but smaller samples.
Analytical perspective	Healthcare‐system, societal, and provider/payer perspectives	Most studies use a healthcare‐system viewpoint; about one‐third include societal costs.
Population type	Chronic HF, HFrEF, and HFpEF populations	Older adults predominate (mean age ≈ 70–78 years); few analyses for HFpEF or younger cohorts.
Sample size	Typically ranges from several hundred to >1 million patients	Large national registry studies expand evidence scope; trial‐based studies involve fewer participants.
Follow‐up duration	Short (<1 year), intermediate (1–3 years), and long (>3 years)	One‐year cost horizons most common across studies.
Setting	Hospital‐based and community/mixed settings	Hospital‐centred data dominate; community and primary‐care costs underrepresented.
Cost components reported	Hospitalization, outpatient care, medication, indirect/societal costs	Hospitalization data nearly universal; indirect costs less consistently captured.

Complete study list (*n* = 49) and detailed variables are provided in *Table* S1.

HF, heart failure; HFpEF, heart failure preserved ejection fraction; HFrEF, heart failure reduced ejection fraction.

### Resource use

Thirty‐six studies identified in the literature search and included in the review provided resource use data (*Table* *3*, *Table* S2).^14^, ^15^, ^16^, ^17^, ^18^, ^19^, ^20^, ^21^, ^22^, ^23^, ^24^, ^25^, ^26^, ^27^, ^28^, ^29^, ^30^, ^31^, ^32^, ^33^, ^34^, ^35^, ^36^, ^37^, ^38^, ^39^, ^40^, ^41^, ^42^, ^43^, ^44^, ^45^, ^46^, ^47^, ^48^, ^49^ A comprehensive investigation of the literature was conducted to identify and analyse resource utilization associated with patients living with HF. The varied settings, interventions, sub‐populations and resource categories analysed in the studies made direct comparisons of resource use challenging. Additionally, variations in health systems across countries likely contributed to some of the heterogeneity in the results.

**Table 3:** Studies reporting resource use associated with HF treatment (condensed)

Parameter	No. of studies	Typical range/median	Main findings
Hospitalizations (HF‐related)	28	0.5–1.3 admissions per patient per year	Primary resource driver; decrease of 15%–50% with DMPs/telemonitoring
Length of Stay (days)	25	6–14 (mean)	Reduced by 3–6 days under multidisciplinary care
Emergency visits	10	0.3–0.8 per patient per year	Decrease of >30% in DMP vs usual care
Outpatient/GP visits	12	3–8 per 6 months	Slight increase with intensive programmes but reduced hospital admissions
Telemonitoring contacts	5	80–90 outbound calls per 100 patients per week	Maintains stability; readmissions reduced by 20%–40%
Medication use	>20	ACEI 70%–90%; beta‐blockers 60%–80%	Optimized therapy linked to lower LOS/readmissions
Nursing/home visits	8	2–5 per month per patient	Increased staff time offset by reduced hospital days

Complete data by country and study design is reported in *Table* S2.

ACEI, ACE inhibitors; DMP, disease management programme; GP, general practitioner; HF: heart failure.

#### General characteristics of included studies

The studies included retrospective and prospective database analyses as well as clinical trials. The review encompassed data collected from the years 1995^26^, ^27^ to 2019,^15^ with follow‐up periods ranging from 3 months^49^ to 5 years.^16^ Some articles compared resource utilization for routine interventions, including additional scheduled visits and evaluations^21^, ^23^, ^30^, ^35^, ^38^, ^39^, ^41^, ^44^, ^45^, ^46^, ^48^ or telemonitoring.^25^, ^37^, ^40^ In other cases, studies compared the addition of a drug to the therapy with a placebo^15^, ^24^, ^28^, ^29^ or a lower dose.^31^ Other comparisons included HF patients versus those without HF,^17^, ^34^, ^36^, ^43^ hospitalized HF patients previously diagnosed with HF versus those not diagnosed,^19^ resource utilization before and after a HF diagnosis,^32^ and patient follow‐up using electronic scales with telephone contact.^42^

#### Resource use parameters

**Figure 2:**
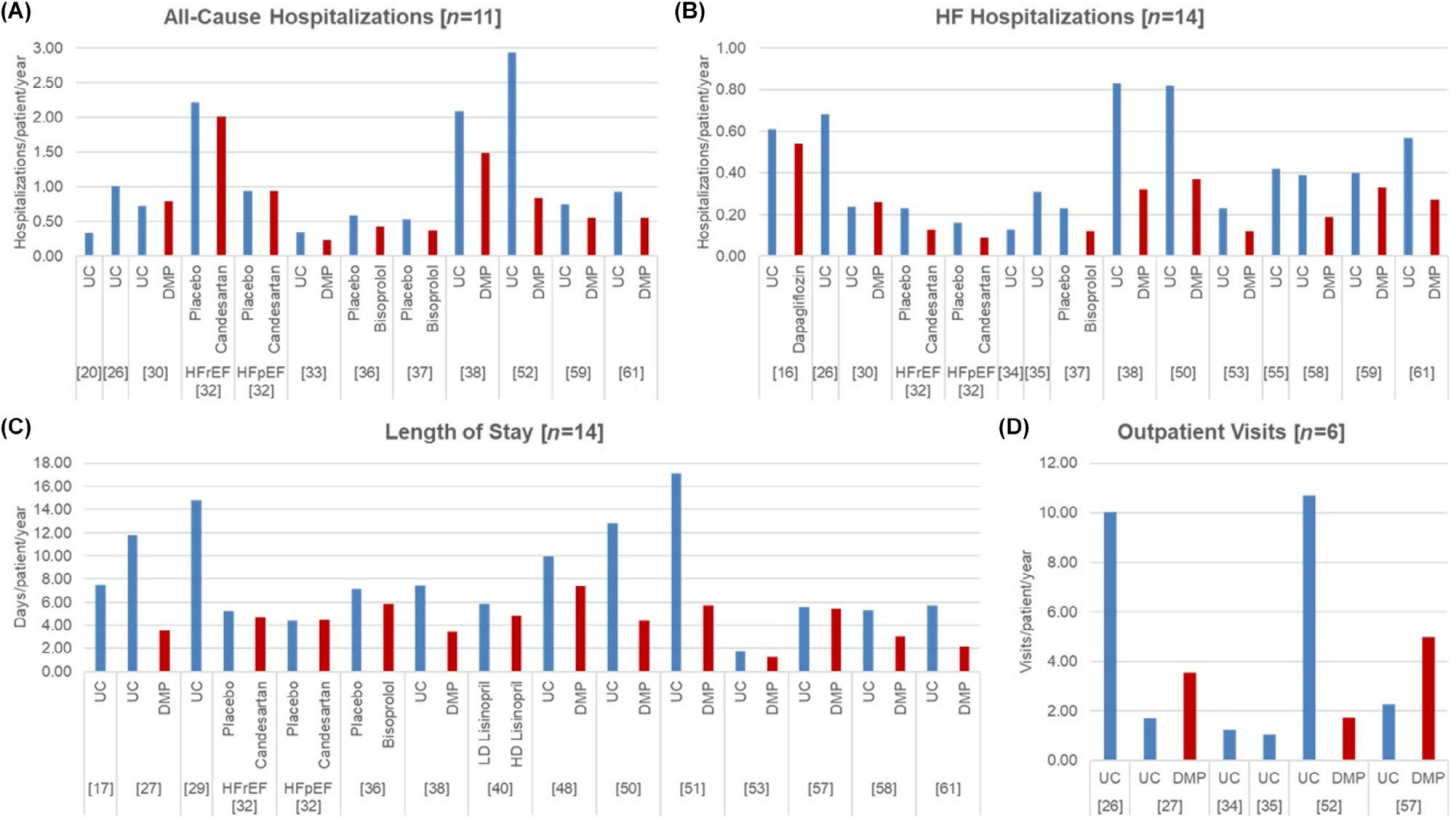
Bar graphs illustrating resource utilization among HF patients: (A) hospitalizations due to any cause, (B) hospitalizations specifically due to worsening HF, (C) total days of hospitalization for any cause, and (D) outpatient visits. ‘UC’ refers to patients managed according to standard clinical practice. ‘DMP’ refers to patients receiving structured interventions designed to optimize HF treatment,^46^ including approaches such as intensive patient management,^21^ IS,^23^, ^44^ home‐based telecardiology,^25^ specialist nurse intervention,^30^ telemedicine,^37^ PREFER,^38^, ^39^ telemonitoring,^40^ telephone contact^42^ and intensive follow‐up.^45^, ^48^ DMP, disease management programme; HD, high dose; HF, heart failure; HFpEF, heart failure preserved ejection fraction; HFrEF, heart failure reduced ejection fraction; IS, intensive support; LD, low dose; PREFER, Palliative advanced home caRE and heart FailurE caRe; UC, usual care.

Generally, to calculate resource use, the studies employed various measures depending on the research objectives, leading to high heterogeneity in parameters across the studies. Most papers included the number and/or length of hospitalizations due to HF and, in some cases, hospitalizations caused by other cardiovascular diseases or any cause, as well as the number of readmissions (*Figure* *2* *A–C*).^14^, ^15^, ^16^, ^18^, ^20^, ^21^, ^22^, ^23^, ^24^, ^25^, ^26^, ^27^, ^28^, ^29^, ^30^, ^31^, ^32^, ^35^, ^37^, ^38^, ^39^, ^40^, ^41^, ^42^, ^44^, ^45^, ^46^, ^47^, ^48^, ^49^, ^50^

Another commonly analysed parameter was medication, including drugs prescribed for HF such as angiotensin converting enzyme inhibitors (ACEI), beta‐blockers, angiotensin receptor blockers (ARBs) or diuretics.^14^, ^15^, ^16^, ^17^, ^19^, ^21^, ^23^, ^26^, ^27^, ^30^, ^33^, ^34^, ^36^, ^37^, ^41^, ^42^, ^43^, ^47^, ^48^ Additional resources, measured by at least two or more studies, included outpatient or ambulatory appointments (*Figure* *2* *D*),^20^, ^21^, ^26^, ^27^, ^39^, ^44^, ^47^ primary care visits,^16^, ^32^, ^35^, ^47^ general practitioner visits,^26^, ^27^, ^32^, ^38^, ^47^ emergency room visits,^15^, ^16^, ^22^, ^35^ intensive care unit utilization,^18^, ^20^, ^22^, ^28^, ^47^ phone contacts,^21^, ^35^, ^39^, ^44^, ^46^, ^47^ home visits,^20^, ^32^, ^35^, ^39^, ^44^, ^47^ physician visits,^35^, ^39^ nursing services,^32^, ^35^, ^39^, ^46^, ^47^, ^49^ laboratory tests (e.g., blood analysis, potassium/creatinine levels and NT‐proBNP tests),^16^, ^20^, ^33^ diagnostic or follow‐up tests [e.g., blood pressure monitoring, echocardiograms, cardiac magnetic resonance images (MRIs) and ECGs],^20^, ^33^ and procedures (e.g., coronary angiography, balloon angioplasty, stent implantation, coronary artery bypass grafting and valve procedures).^20^, ^24^

#### Heart failure management programmes

The results regarding resource use demonstrated that HF patients require significant resources, particularly for hospitalizations and emergencies. Consequently, 16 articles compared various disease management programmes to identify which utilized fewer resources.^21^, ^23^, ^25^, ^30^, ^35^, ^37^, ^38^, ^39^, ^40^, ^41^, ^42^, ^44^, ^45^, ^46^, ^48^ These programmes were defined as usual care,^21^, ^23^, ^25^, ^30^, ^35^, ^37^, ^38^, ^39^, ^40^, ^41^, ^44^, ^45^, ^46^, ^48^ intensive patient management,^21^ nurse‐led multidisciplinary care,^21^ basic support,^23^, ^44^ intensive support,^23^, ^44^ telemonitoring or telephone contact,^25^, ^37^, ^40^, ^42^ specialist nurse intervention,^30^ PREFER (Palliative advanced home caRE and heart FailurE caRe),^38^, ^39^ onsite HF service (in the context of elderly care homes),^41^ electronic scales^42^ and intensive follow‐up.^45^, ^48^

Care programmes providing enhanced support to HF patients generally reduced the number and duration of hospitalizations, as well as primary care and emergency room visits related to HF (*Figure* *2* *A–C*).^21^, ^23^, ^25^, ^30^, ^35^, ^37^, ^38^, ^39^, ^40^, ^45^, ^46^, ^48^ However, some studies noted that disease management programmes often required more hours from healthcare professionals, such as GPs, cardiologists and nurses, due to the need for closer patient monitoring and treatment.^35^, ^38^, ^46^ For example, the PREFER programme required 8.2 h of GP services and 66.1 h of other medical professionals' time per patient over 6 weeks, whereas usual care required 4.0 and 6.6 h per patient, respectively. However, this increase was offset by a reduction in hospital care duration, with PREFER patients requiring 2.9 days per patient compared with 8.6 days per patient under usual care.^38^ Other programmes incorporated rehabilitation services,^35^ additional time for telemonitoring, and extra visits compared with usual care.^46^

#### Effect on resource use of drug additions to heart failure therapy

Clinical trials evaluating new medicines for HF therapy have demonstrated a reduction in hospital admissions (*Figure* *2* *A,B*).^15^, ^24^, ^28^, ^29^, ^31^ The reduction was estimated from 6.8%^24^ to 42.7%.^29^ The drugs assayed were dapagliflozin,^15^ candesartan,^24^ bisoprolol^28^, ^29^ and lisinopril.^31^ These medications were prescribed daily, and patients enrolled in these studies were followed for a mean duration of 12 to 46 months.

#### Resources use of HFrEF and HFpEF patients

Just one article reported resource use in HF reserved ejection fraction (HFrEF) patients and HF preserved ejection fraction (HFpEF).^51^ The management of these patients involved significant outpatient visits, medication adjustments and hospital readmissions with some differences between HFrEF and HFpEF patients.

Over the 3‐month study follow‐up, the rate of unscheduled visits was similar between HFrEF and HFpEF patients, at 0.46 and 0.49 visits per patient, respectively. Patients with HFrEF had slightly more scheduled visits (3.5 visits per patient) compared with HFpEF patients (3.2 visits per patient). As a result, HFrEF patients required more clinic time, with an average of 135 min per patient compared with 125 min for HFpEF patients.

In terms of telemonitoring, both HFrEF and HFpEF patients had similar frequencies of inbound and outbound calls. For inbound calls, HFrEF patients averaged 16.9 calls per 100 patients per week, while HFpEF patients averaged 16.1 calls. For outbound calls, the rates were 84.9 calls per 100 patients per week for HFrEF and 81.9 for HFpEF.

Medication adjustments were a key aspect of the clinical workload, particularly with diuretics and other heart failure medications. Around 92% of patients were prescribed diuretics, with approximately 45% requiring at least one dose change during the 3‐month programme. The frequency of diuretic dose adjustments was similar for both HFrEF and HFpEF patients. Additionally, HFrEF patients were more frequently prescribed ACE inhibitors and beta‐blockers, with more dose changes compared with HFpEF patients.

Hospital readmission rates were slightly higher for HFpEF patients compared with HFrEF patients. Specifically, the readmission rate for HFpEF was 0.99 admissions per patient over 12 months, while for HFrEF, it was 0.86 admissions per patient. The rate of emergency cardiovascular readmissions was similar for both groups, at 0.31 admissions per patient for HFrEF and 0.28 for HFpEF. However, emergency non‐cardiovascular readmissions were notably higher for HFpEF patients, with a rate of 0.46 admissions per patient compared with 0.31 for HFrEF patients.

### Costs of heart failure

Twenty‐six records identified in the literature search and included in the review provided data on the financial burden of HF (*Table* *4*, *Table* S3).^15^, ^16^, ^17^, ^18^, ^19^, ^20^, ^24^, ^25^, ^29^, ^32^, ^35^, ^37^, ^38^, ^44^, ^45^, ^46^, ^47^, ^48^, ^49^, ^51^, ^52^, ^53^, ^54^, ^55^, ^56^, ^57^, ^58^, ^59^ A comprehensive literature search was conducted to identify and analyse both direct and indirect expenses due to HF. The heterogeneity of cost studies, due to differences in experimental designs, made the comparison of economic data challenging.

**Table 4:** Costs associated with HF treatment (condensed)

Cost category	No. of studies	Range (€ per patient per year)	Median share of total cost (%)	Key observations/trends
Total annual cost (all components)	26	613–22,647	100	Wide variation across studies due to heterogeneity in methodology, healthcare systems, and included cost components.
Hospitalization	25	1500–18,500	60–92	Dominant cost driver in almost all studies; reductions of 20%–40% reported with disease‐management or telemonitoring interventions.
Outpatient/Primary‐care visits	15	100–1400	5–20	Smaller but consistent contributor; higher frequency of visits often associated with lower hospital use.
Medication	18	200–1100	8–18	Costs stable across settings; pharmacotherapy intensity higher in HFrEF.
Emergency‐department care	8	50–500	< 5	Lower share of total cost; reduced under structured follow‐up models.
Diagnostic/Monitoring procedures	10	100–800	5–10	Mainly echocardiography, laboratory, and device checks; higher costs in advanced disease stages.
Indirect/Societal costs	6	400–12,700	Variable	Include productivity loss and informal care; substantial in younger or community‐dwelling populations.

HF, heart failure; HFrEF, heart failure reduced ejection fraction.

The articles included data from retrospective and prospective database analyses, clinical trials and one therapeutic positioning report, with data collected between 1998^49^ and 2020.^52^ Follow‐up periods ranged from 3 months^49^ to 5 years,^16^ although one study calculated costs over a 20‐year time horizon.^57^

#### Country‐specific estimates

**Figure 3:**
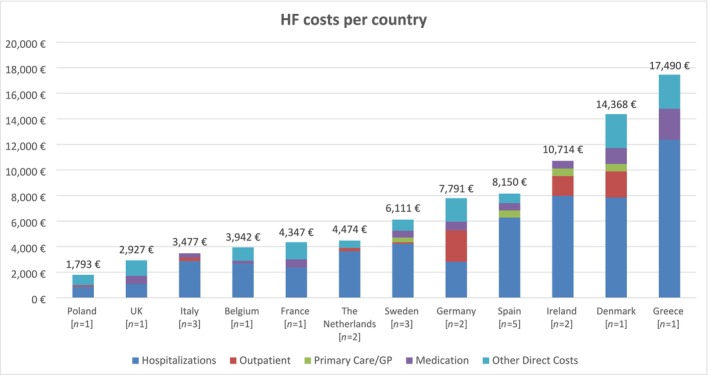
Bar graph displaying the average annual HF‐related cost per patient across European countries. Values represent the mean cost estimates reported in the included studies divided in five cost categories: hospitalizations, outpatient, primary care and general practitioner, medication and other direct costs. GP, general practitioner; HF, heart failure; PC, primary care.

Data on costs per HF patient from 12 European countries were obtained and compared (*Table* S4, *Figure* *3*). Costs ranged from €613 per patient per year in Italy^25^ to €22,647 per patient per year in Germany.^55^ Notably, there was considerable variability between studies conducted in the same country, with differences of nearly €19,000 in Spain and €20,000 in Germany between the minimum and maximum reported values.

#### Heart failure management programmes

Seven articles aimed to compare the economic burden of different treatment procedures. These studies analysed usual care versus disease management programmes that included additional visits and intensive monitoring (*Figure* S1).^35^, ^38^, ^44^, ^45^, ^46^, ^48^, ^49^ Six articles concluded that usual care imposed a greater economic burden compared with more intensive healthcare support.^35^, ^38^, ^45^, ^46^, ^48^, ^49^ However, one study found that usual care—consisting of routine follow‐ups with a cardiologist—had a similar cost to basic support, which included patient education, counselling on heart HF, visits to an HF nurse, and telephone contact. Additionally, usual care was found to be slightly less expensive than intensive support, which incorporated additional HF nurse visits, home nurse visits, 24‐h telephone availability and multidisciplinary advice.^44^ This outcome is attributed to the fact that, although these programmes often require at least one physician and one nurse to follow up participating patients, the costs associated with visits (either at health centres or at home) and phone calls are offset by a significant reduction in hospitalization costs.

Other records compared usual care with telemonitoring programmes in Italy,^25^ the Netherlands,^57^ and Spain.^37^ Two studies concluded that telemonitoring reduced healthcare costs.^25^, ^37^ Instead, the third study estimated that two different modalities of telemonitoring were more expensive than usual care.^57^

#### Cost analysis of drug additions to heart failure therapy

The costs associated with adding a new drug to heart failure (HF) therapy were examined in three studies using data from clinical trials.^15^, ^24^, ^29^

The first trial studied dapagliflozin. Clinical trials were conducted in the United Kingdom, Germany and Spain on patients with Heart Failure with reduced Ejection Fraction (HFrEF). The results indicated that incorporating this drug into HF therapy increased annual costs per patient across all countries, primarily due to expenses related to treatment, monitoring and adverse events.^15^

Clinical trials evaluating candesartan were conducted in France, Germany and the United Kingdom, including patients with both HFrEF and Heart Failure with preserved Ejection Fraction (HFpEF), who were divided into subgroups. In France, the addition of candesartan was cost‐saving for the HFrEF subgroup, whereas in other subgroups, costs showed a slight increase. Overall, the cost of candesartan was substantially offset by a reduction in hospitalizations, especially for HF.^24^

Regarding bisoprolol, the clinical trial focused exclusively on patients with HFrEF. The findings estimated that while overall lifetime costs were higher for patients receiving bisoprolol, hospitalization‐related costs per patient were reduced.^29^

However, as discussed in Cost‐effectiveness studies on clinical trials section, these studies also included cost‐effectiveness analyses, providing a more comprehensive perspective on the benefits these drugs offer to patients.

#### Hospitalization costs

To estimate HF costs, most studies focused on hospitalization or inpatient admission costs due to HF.^16^, ^17^, ^18^, ^19^, ^20^, ^22^, ^24^, ^25^, ^29^, ^35^, ^38^, ^44^, ^45^, ^46^, ^47^, ^48^, ^49^, ^51^, ^54^, ^55^, ^57^ The contribution of hospitalization to total costs ranged from 15%^54^ to 92%.^19^ Some articles comparing disease management programmes considered only hospitalization charges for usual care, assuming that other costs, such as medication, would be similar between groups.^25^, ^45^, ^46^, ^48^, ^49^ Other studies, however, separated costs related to emergencies^16^, ^35^, ^38^, ^53^, ^54^, ^55^, ^57^ and ICU use.^20^, ^24^, ^47^

Despite this, most references identified hospitalization costs as the primary driver of the economic burden of HF.^16^, ^17^, ^18^, ^19^, ^22^, ^24^, ^35^, ^38^, ^44^, ^47^, ^51^, ^55^ The estimated contribution of hospitalization to total costs under usual care ranged from 37.0%^24^ to 91.7%.^19^

Studies comparing intensive support programmes and telemonitoring with usual care demonstrated that these interventions significantly reduce hospitalization costs.^25^, ^35^, ^38^, ^44^, ^45^, ^46^, ^48^, ^49^, ^57^ The estimated cost reductions varied widely, from a maximum reduction of 91%^49^ to more moderate reductions of 23.7%.^25^ Interestingly, one study even estimated a slight increase in hospitalization costs for intensive support programmes, at 4.3%.^44^

#### Primary care costs

Nine studies considered the financial costs of primary care management for HF patients, including costs associated with GPs.^16^, ^17^, ^32^, ^35^, ^38^, ^47^, ^51^, ^54^, ^57^ Estimates of the contributions of primary care to the overall economic burden ranged from 1.1%^54^ to 22%.^47^ The total costs per patient per year in Europe were estimated to range from €203.71^54^ to €1246.^35^

#### Other healthcare costs

Medication was considered in 14 studies, typically including drugs prescribed for conditions other than HF.^16^, ^17^, ^18^, ^19^, ^20^, ^22^, ^24^, ^29^, ^35^, ^47^, ^52^, ^53^, ^54^, ^55^ One paper estimated that out of a total of €1083 per patient spent on medication, €417 was specifically attributed to drugs prescribed for HF, a 40% approximately.^16^

Six studies assessed outpatient facility costs, referring to expenses associated with visits.^17^, ^19^, ^20^, ^44^, ^47^, ^55^

Ten studies estimated the costs of procedures and laboratory tests, which were sometimes analysed together.^16^, ^18^, ^19^, ^20^, ^22^, ^24^, ^32^, ^44^, ^53^, ^55^ Menafoglio et al. provided details on the costs of non‐invasive procedures, such as echocardiograms or ECGs,^53^ while McMurray et al. reported economic data on surgical procedures, including catheterizations and heart transplants.^24^

Costs associated with medical professionals other than GPs were considered in eight studies.^17^, ^18^, ^32^, ^38^, ^47^, ^48^, ^49^, ^54^ These articles often focused on costs related to nursing, either in primary care^32^, ^48^ or at secondary care.^47^, ^49^, ^54^ Other professionals mentioned included cardiologists or specialists,^48^, ^54^ psychiatrists,^17^ dieticians,^49^ and paramedical staff.^49^ General terms such as ‘physicians’^18^ or ‘other medical professionals’^38^ were also reported. Finally, some studies included costs related to formal care provided by a caregiver^17^, ^35^, ^54^ and transportation expenses.^35^, ^38^, ^54^

#### Indirect costs

Three studies provided information on the indirect costs incurred by patients.^16^, ^17^, ^54^ The financial burden associated with indirect costs due to HF varied significantly across these estimations. The lowest cost was €44.40 per patient per year, attributed to sick leave.^16^ Another paper reported indirect costs of €2726, due to foregone earnings.^17^ The highest estimation of indirect costs was related to informal, non‐professional care provided by relatives. This was assessed using the proxy good method, which evaluates the time devoted to caregiving based on the market price of a comparable professional service. The estimated costs of informal care ranged from €7683 to €12,723 (representing 59.1% to 69.8% of the total cost), depending on the scenario assumed.^54^

#### Co‐morbidities

HF patients are typically older and often have co‐morbidities that can exacerbate their condition, leading to increased treatment costs. For this reason, some studies included in the review provided data on patients with additional conditions that could contribute to higher hospitalization and medication expenses. Cardiovascular‐related co‐morbidities reported in at least three studies included hypertension,^17^, ^18^, ^19^, ^21^, ^32^, ^33^, ^36^, ^39^, ^45^, ^46^, ^48^ atrial fibrillation,^16^, ^17^, ^18^, ^19^, ^21^, ^32^, ^33^, ^39^, ^45^ myocardial infarction,^17^, ^21^, ^32^, ^33^, ^36^, ^45^, ^46^ ischemic heart disease,^16^, ^17^, ^33^, ^39^, ^45^, ^48^ stroke,^16^, ^17^, ^19^, ^21^, ^39^, ^45^ and peripheral vascular disease.^16^, ^18^, ^19^, ^32^

Other co‐morbidities commonly reported included diabetes mellitus,^16^, ^17^, ^18^, ^19^, ^21^, ^32^, ^33^, ^36^, ^39^, ^45^, ^46^, ^48^ chronic obstructive pulmonary disease (COPD),^16^, ^17^, ^21^, ^32^, ^36^, ^45^, ^46^ chronic kidney disease,^16^, ^17^, ^18^, ^32^, ^33^, ^39^ depression,^18^, ^32^, ^36^, ^39^ and anaemia.^18^, ^32^, ^45^

Additionally, some studies analysed the costs associated with these co‐morbidities. Co‐morbid conditions accounted for 26.7% to 32.7% of hospitalization costs among HF patients.^16^, ^20^ One study estimated that 61.6% of total medication costs were attributed to co‐morbidities.^16^

#### Costs of HFrEF and HFpEF patients

The total costs incurred during the first year of treatment for HFrEF and HFpEF patients reflect the extensive resource utilization required for their management. The average total annual cost per patient is higher for HFrEF patients at €13,011 compared with €12,206 for HFpEF patients (*Figure* S2).^51^ These figures encompass expenses related to hospital admissions, outpatient care, medication and disease management. While costs for HFrEF patients are primarily driven by the initial hospitalization and structured outpatient follow‐ups, the cost burden in HFpEF patients is significantly influenced by a higher rate of emergency non‐cardiovascular readmissions.

Emergency non‐cardiovascular admissions represent a major component of the overall financial burden for both groups. HFpEF patients experience higher costs per emergency non‐cardiovascular admission, with an average expense of €655, compared with €584 for HFrEF patients.^51^ This is in line with previous findings highlighting the greater prevalence of non‐cardiac co‐morbidities in HFpEF, leading to increased healthcare utilization beyond heart failure‐specific care.^15^

Intensive outpatient disease management is required for both HFrEF and HFpEF patients, particularly in the first 3 months following discharge. The associated costs during this period amount to €791 for HFrEF patients and €693 for HFpEF patients.^51^ These expenses reflect the structured care needed to optimize medical therapy, including scheduled and unscheduled clinic visits as well as telephone consultations.

Medication and general practitioner visit costs also contribute significantly to the total expenditure. HFrEF patients have higher medication costs of €596 compared with €527 in HFpEF patients, consistent with the broader use of evidence‐based pharmacological treatments in HFrEF.^51^ Similarly, general practitioner visits cost €594 for HFrEF patients and €569 for HFpEF patients.^51^ These differences reflect the greater frequency of medication titration and follow‐up required for patients with HFrEF.

### Cost‐effectiveness

Six analyses regarding cost‐effectiveness of HF programmes or drugs on clinical assays were included in the SLR (*Table* S5).^15^, ^24^, ^29^, ^35^, ^38^, ^57^ The cost‐effectiveness analyses collected data between 2001^29^ and 2020.^15^ Study periods ranged from 6 months^29^ to a 20‐year time horizon.^57^

#### Heart failure management programmes

Three articles conducted cost‐effectiveness analyses to determine the efficacy of different treatment procedures.^35^, ^38^, ^57^

González‐Guerrero et al. estimated that a disease management programme for older HF patients (mean age of 85 years) demonstrated a 91% and 85% probability of being cost‐effective from healthcare and societal perspectives, respectively, with a cost of €44,000 per Quality Adjusted Life Year (QALY).^35^

Meanwhile, Sahlen et al. found that HF patients (mean age of 81.9 years) receiving palliative advanced home care and HF care intervention gained 0.25 QALYs, alongside a cost reduction of €50,000.^38^

Finally, the last article described a cost‐effectiveness analysis comparing usual care with two different programmes: home telemonitoring and nurse telephone support. While both programmes had higher costs per patient than usual care (€27,186 for home telemonitoring, €24,604 for nurse telephone support, and €14,414 for usual care), they were more effective in managing patients, with QALY values of 2.93, 3.07 and 1.91, respectively. Consequently, nurse telephone support was considered cost‐effective at a willingness‐to‐pay (WTP) threshold of €9000/QALY or higher, and home telemonitoring at a WTP threshold of €14,000/QALY or higher compared with usual care.^57^

#### Cost‐effectiveness studies on clinical trials

The cost‐effectiveness of adding a new drug to HF therapy was investigated in three articles using data from clinical trials.^15^, ^24^, ^29^ The three cost‐effectiveness studies included only HF patients with HFrEF. The drugs studied were dapagliflozin,^15^ candesartan,^24^ and bisoprolol.^29^

For dapagliflozin, the incremental cost‐effectiveness ratios were €6828, €5379 and €9406 per QALY in the United Kingdom, Germany and Spain, respectively. Additionally, more than 90% of probabilistic sensitivity analysis simulations indicated cost‐effectiveness at a WTP threshold of €23,457/QALY in the United Kingdom (equivalent to £20,000/QALY) and €20,000/QALY in Germany and Spain.^15^

In the cost‐effectiveness analysis for candesartan, results show that this drug was cost‐saving in certain scenarios, while in others, the maximum cost per life year gained was €3881.^24^

Regarding bisoprolol, when the costs of added years of life were excluded, the drug increased life expectancy at an incremental cost of €1490 per life year gained. However, when these costs were included, the incremental cost‐effectiveness ratio for bisoprolol therapy rose to €19,216 per life year gained.^29^

## Discussion

This comprehensive SLR provides valuable insights into the financial burden of HF in Europe. While most studies examining HF resource utilization reported data on drug prescriptions^14^, ^15^, ^16^, ^17^, ^19^, ^21^, ^23^, ^26^, ^27^, ^30^, ^33^, ^34^, ^36^, ^37^, ^41^, ^42^, ^43^, ^47^, ^48^ and the number and duration of hospitalizations due to HF,^14^, ^15^, ^16^, ^18^, ^20^, ^21^, ^22^, ^23^, ^24^, ^25^, ^26^, ^27^, ^28^, ^29^, ^30^, ^31^, ^32^, ^35^, ^37^, ^38^, ^39^, ^40^, ^41^, ^42^, ^44^, ^45^, ^46^, ^47^, ^48^, ^49^ only a few articles quantified other parameters, such as laboratory tests,^16^, ^20^, ^33^ diagnostic or follow‐up tests,^20^, ^33^ and surgical procedures.^20^, ^24^

Studies generally concluded that costs associated with HF hospitalizations are the primary contributors to the economic burden of the disease, accounting for 15%^54^ to 92%^19^ of total costs. Hence, several studies focused on reducing medical expenses related to hospitalizations by implementing alternative monitoring methods for patient management.^25^, ^35^, ^37^, ^38^, ^44^, ^45^, ^46^, ^48^, ^49^, ^57^ In comparison, the contribution of primary care to HF's economic burden was estimated to range between 1.1%^54^ and 22%.^47^ Nevertheless, a comparison of overall HF costs across all included studies revealed significant variability, with estimates ranging from €613^25^ to €22,647^55^ per patient per year for usual care.

In Europe, the economic burden of managing HF is estimated to account for 1% to 7% of the total healthcare expenditure.^56^, ^60^ Direct costs associated with inpatient care include various components such as medication, laboratory services, doctors' fees, ICUs and nursing home expenses. These expenditures are directly tied to the delivery of medical services within a hospital setting. In contrast, outpatient care involves costs related to hospital outpatient services, doctors' consultation fees, specialized medical services, home care, medication, laboratory tests, medical procedures, paramedical personnel and medical transport. Together, these outpatient costs underscore the breadth of healthcare services provided outside the hospital, highlighting the comprehensive scope of healthcare expenses associated with both inpatient and outpatient HF care.^56^, ^61^

Indirect costs associated with HF encompass multiple components. A key factor is caregiver costs, which are calculated by multiplying the average annual number of inpatient days attributable to HF by the average daily market price for caregiver services. This captures the economic impact on caregivers assisting HF patients.^61^ Additionally, indirect costs include productivity losses caused by morbidity and mortality among individuals below retirement age, as longer hospital stays and co‐morbidities have been related to work detachment.^17^ These costs are often estimated using mathematical models based on the human capital approach, which assigns an economic value to productivity losses due to health‐related issues. Collectively, indirect costs offer a broader perspective on the economic burden of HF, reflecting both caregiver expenses and productivity losses.^61^

The differences in findings can be attributed to the substantial heterogeneity in the objectives, methodologies and outcomes of the studies. Additionally, some studies compared resource use and costs for various interventions, such as the addition of a drug to the therapy^15^, ^24^, ^28^, ^29^, ^31^ or the implementation of a patient management programme.^21^, ^23^, ^25^, ^30^, ^35^, ^37^, ^38^, ^39^, ^40^, ^44^, ^45^, ^46^, ^48^, ^49^, ^57^

A source of variation arose from the different populations included in the studies. Trials often included patients with shared characteristics to minimize variability in results. For example, some studies only included patients with HFrEF,^15^, ^29^, ^52^ while others considered HF patients across different New York Heart Association (NYHA) functional classes.^21^, ^24^, ^29^, ^38^, ^39^, ^40^, ^42^, ^45^, ^62^ Murphy et al. compared HF costs between HF patients with HFpEF and HFrEF, demonstrating cost differences between these groups.^51^ Consequently, differences in patient populations likely contribute to the variability in findings. Moreover, the size of the patient cohort also influences results; while some studies analyse national databases with data from entire countries, others were limited to smaller patient groups. The smallest study included 30 HF patients,^41^ whereas Neumann et al. examined the economic burden of all HF patients in Germany over a seven‐year period, totalling 1,926,710 HF diagnoses.^55^

Some studies excluded certain costs, deeming them irrelevant to their specific research focus. For instance, many studies comparing intensive support or telemonitoring with usual care considered only hospitalization costs, while medication and primary care costs were excluded.^25^, ^37^, ^44^, ^45^, ^46^, ^48^, ^49^ This includes the study that estimated lower costs per patient due to HF; however, since most costs were omitted from this calculation, the estimation primarily reflects expected cost reductions.^25^ Similarly, González‐Loyola et al. sought to estimate the overall economic burden of HF in a primary care setting but did not account for hospitalization costs.^32^

There is also significant uncertainty regarding the contribution of indirect costs to the overall economic burden of HF. Only three studies assessed indirect costs, yielding widely divergent estimates, ranging from €44.40 to €12,723 per patient per year.^16^, ^17^, ^54^ Additionally, Cook et al. estimated that indirect costs accounted for ~40% of the global HF economic burden.^60^

In some cases, implementing a disease management programme or introducing a new drug increased the economic burden of HF treatment. However, cost‐effectiveness studies indicated that these costs could be offset by improvements in patients' life expectancy and quality of life.^15^, ^24^, ^29^, ^35^, ^38^, ^57^

Generally, programmes that provided greater support to patients than usual care or introduced telemonitoring reduced hospitalization costs and resource use, indicating that such programmes can result in budget savings that could be reallocated to other purposes such as improving risk stratification and diagnosis of HF. Additionally, the development of more reliable diagnostic tools that improve patient risk stratification may increase this effect. This highlights the potential of novel technologies, such as artificial intelligence, in healthcare systems to help reduce the financial burden of HF and, in turn, deliver broader benefits to society.

This SLR compiled various studies estimating the resource use and financial burden of acute and chronic HF on European healthcare systems. The research explores the resources required to treat HF in diverse scenarios, identifying key drivers of expenditure and comparing approaches to reduce this burden. Typically, HF patients are older and present with multi‐morbidities, complicating the accurate estimation of HF‐related costs and worsening prognostics.^8^ However, this SLR noted that some studies successfully distinguished resources utilized specifically for HF treatment from those used for managing other conditions.

Despite these efforts, weaknesses in cost estimation were identified, particularly regarding indirect costs. Informal care and/or productivity losses were rarely assessed, and the few available estimates varied widely, reflecting uncertainty about their contribution to the overall economic burden. Additionally, the high variability in methodologies and outcomes across studies poses challenges to providing precise HF cost estimates for individual countries, even though treatment procedures should be similar across ESC member countries. Furthermore, the review acknowledges the overrepresentation of certain European regions, with most studies conducted in Western and Southern Europe. Another limitation is that the review only includes articles written in English and Spanish.

This study offers an extensive collection of European health economic research on HF, providing a platform for comparing diverse perspectives on the topic. It identifies common trends while highlighting significant variability in HF cost estimations across studies. Future research should consider these findings to critically evaluate how healthcare systems can be improved to enhance treatment efficacy. This information can guide HF researchers in understanding the impact of HF on European healthcare systems and assist policymakers in effectively prioritizing resource allocation.

HF imposes a significant burden on our healthcare systems and society. Numerous studies have identified hospitalization as the primary cost driver, highlighting the potential to reduce medical expenses through alternative treatments, advanced monitoring methods and improved risk stratification in patient management. Continued research into innovative therapies is essential for improving patients' quality of life while alleviating the financial strain associated with this condition. Furthermore, future updates to national and international guidelines should emphasize strategies to shorten the time to diagnosis, ensuring timely and effective care.

## Conflict of interest

The authors affirm that they do not have any competing interests.

## Funding

The study received funding from the European Union's Horizon Europe research and innovation programme under grant agreement number 101080905, UK Research and Innovation grant award with reference number 10073472, State Secretariat for Education, Research and Innovation (SERI), Federal Department of Economic Affairs, Education and Research (EAER), and Finance Research and Innovation/European Framework Programmes, for Suisse Confederation. The funders had no role/influence in the design of the study, prospective data collection, analyses, interpretation of data and drafting of the manuscript. R.J.S. is supported by the National Institute for Health and Care Research (NIHR) Newcastle Biomedical Research Centre (BRC). The NIHR Newcastle BRC is a partnership between Newcastle Hospitals NHS Foundation Trust, Newcastle University and Cumbria, Northumberland, and Tyne and Wear NHS Foundation Trust and is funded by the NIHR. The views expressed are those of the author(s) and not necessarily those of the NIHR or the Department of Health and Social Care.

## Supporting information


**Table S1.** General characteristics of studies selected.
**Table S2.** Studies reporting resource use associated with HF treatment.
**Table S3.** Studies reporting costs associated with HF treatment.
**Table S4.** Heart failure costs for usual care in some European countries.
**Table S5.** Cost‐effectiveness analyses regarding heart failure disease management programs and drugs in clinical assays.

**Figure S1.** Bar graphs comparing annual HF‐related costs per patient under UC versus DMP. UC denotes standardized clinical management, while DMP includes structured interventions such as home‐based telecardiology [33], DMP [48,59], telemedicine [50], PREFER [51], IS [57], intensive follow‐up [58,61] and MC [62].

**Figure S2.** Bar graph comparing average annual HF‐related costs per patient between those with HFrEF and HFpEF [21].
