# Supplementary material for: Economic burden of heart failure in Europe: A systematic review of costs and cost‐effectiveness
Source: ESC Heart Fail. 2025 Nov 26;12(6):4055–68. doi: 10.1002/ehf2.70017 (PMC12719840; doi:10.1002/ehf2.70017)
Supplement: Supplementary file 1 — Table S1. General characteristics of studies selected. Table S2. Studies reporting resource use associated with HF treatment. Table S3. Studies reporting costs associated with HF treatment. Table S4. Heart failure costs for usual care in some European countries. Table S5. Cost‐effectiveness analyses regarding heart failure disease management programs and drugs in clinical assays. [file EHF2-12-4055-s003.docx]

**Supplementary Table S1:** General characteristics of studies selected

| **Reference** | **Setting** | **Publication year** | **Type of article** | **Country** | **Data source** | **Population** | **Intervention** | **Comparator** | **Perspective** | **Study description** |  |
| --- | --- | --- | --- | --- | --- | --- | --- | --- | --- | --- | --- |
| Jaarsma T et al. [14] | Resource use | 2005 | Review | The Netherlands | Follow guidelines from ESC | HF patients in the Netherlands | NA | NA | NA | NA |  |
| Informe de Posicionamiento Terapéutico de Dapagliflozina [15] | HF costs | 2022 | Therapeutic Positioning Report | Spain | Costs extracted from published evaluations | HF patients with reduced ejection fraction | Dapagliflozin | Sacubitril-Valsartan, Enalapril,  Valsartan, and placebo | Healthcare system | Cost/year of treatment description |  |
| McEwan P et al. [16] | Resource use  HF costs  Cost-effectiveness | 2020 | Cost-effectiveness analysis | UK  Spain  Germany | Costs extracted from public sources such as NHS and available bibliography | HF patients with reduced ejection fraction (n=8,134) | Dapagliflozin  added to ST | ST | Healthcare system | Markov state-transition cohort model |  |
| Escobar C et al. [17] | Resource use  HF costs | 2020 | Cost-of-illness study | Spain | BIG-PAC database | Patient with HF (n=17,163) | NA | NA | Societal | Observational, retrospective, population-based |  |
| Bundgaard JS et al. [18] | Resource use  HF costs | 2019 | Economic burden estimation | Denmark | Danish nationwide registries | Patients with a first-time hospital-based in- or outpatient diagnosis (n=176,067) | HF patients | Background  population without HF | Societal | Nationwide, retrospective |  |
| Lesyuk W et al. [19] | HF costs | 2018 | SLR of cost-of-illness studies | Germany | Medline, Embase, Scopus, CRD, York Database, NHS EED | HF patients from Spain, Germany, Italy, Poland, Sweden, Greece and  Ireland | Studies  dealing with HF and its economic burden | NA | NA | NA |  |
| Stafylas P et al.  [20] | Resource use  HF costs | 2017 | Economic burden estimation | Greece | Hospitalizations and healthcare resources used collected from the survey | HF patients (n=307) | NA | NA | Social security system | Prospective, observational survey |  |
| Murphy TM et al. [21] | HF costs | 2017 | Cost-of-illness study | Ireland | Casemix data | HF hospitalized survivors in a DMP (n=1,292) | HFrEF | HFpEF | Provider | Retrospective |  |
| Cook C et al. [22] | HF costs | 2014 | Review of cost-of-illness studies | UK (global economic burden estimation) | Records included from MEDLINE, World Bank data | NA | Published economic estimates of healthcare costs attributed to HF | NA | NA | NA |  |
| Menafoglio A et al. [23] | | HF costs | 2014 | Economic burden estimation | Switzerland | Swiss public institutions | Athletes aged 14–35 (n=1,070) | CV evaluation including ECG | CV evaluation  using medical history and physical examination | Healthcare system | Prospective, observational |
| Corrao G et al. [24] | Resource use  HF costs | 2014 | Economic burden estimation | Italy | Health service of Lombardy | Beneficiaries of the Italian NHS hospitalized at least once with a HF diagnosis (n=26,949) | HF cohort | Referent cohort | NHS | Estimation of incidence, outcomes, and costs of HF |  |
| Delgado JF et al. [25] | HF costs | 2014 | Cost-of-illness study | Spain | Spanish Board of Pharmaceutical Associations, Spanish Ministry of Health, Social Services and Equality, Spanish eSalud healthcare cost | Patients with symptomatic HF recruited from specialized cardiology clinics (n=374) | NA | NA | Social | Prospective, multicenter, observational |  |
| Czech M et al. [26] | Resource use  HF costs | 2013 | Cost-of-illness study | Poland | Regulation of the President of the National  Health Fund | HF patients (n=5,275) | NA | NA | Public payer | Program assessing diagnostic procedures |  |
| Berger R et al. [27] | Resource use | 2010 | Pilot study | Austria | STARBRITE study | Patients with clinical signs and symptoms of HF with NYHA functional class III or IV (n=278) | Intensive patient management (n=92) | UC (n=90)  Nurse-Led MC (n=96) | NA | 3-arm, prospective, randomized |  |
| Neumann T et al. [28] | HF costs | 2009 | Economic burden estimation | Germany | German Federal Statistical  Office | Patients diagnosed with HF in Germany from 2000-2007 | NA | NA | Public health system | Analysis of the development of case numbers for HF |  |
| Claes N et al. [29] | Resource use  HF costs | 2008 | Economic burden estimation | Belgium | National hospital  registration system | Patients admitted with HF in Belgium in 2001 | NA | NA | Healthcare system | Retrospective |  |
| Jaarsma T et al. [30] | Resource use | 2008 | Clinical trial | The Netherlands | COACH study | HF patients with ≥18 years (n=1,023) | BS (n=340)  IS (n=344) | UC (n=339) | NA | Multicenter, randomized, controlled trial |  |
| Liao L et al. [31] | HF costs | 2008 | SLR of economic burden estimations | USA (includes data from UK and Sweden) | MEDLINE, EMBASE and  references | NA | Research on HF in the elderly pertaining to costs, resource use, quality of life and cost-effectiveness | NA | NA | NA |  |
| McMurray JJ et al. [32] | Resource use  HF costs  Cost-effectiveness | 2006 | Cost–consequence and cost-effectiveness analysis | Germany  UK  France | CHARM program  Costs from government sources | Patients with NYHA class II–IV HF (n=7,599) | Candesartan added to conventional treatment (n=3,803) | Placebo, i.e. conventional treatment for HF (n=3,796) | France, Germany: third party payer  UK: NHS | Prospective |  |
| Scalvini S et al. [33] | Resource use  HF costs | 2005 | Cost-of-illness study | Italy | NA | HF patients (n=426) | Home-based telecardiology (n=230) | UC (n=230) | Healthcare system | NA |  |
| Laceya L, Tabbererb M [34] | Resource use | 2005 | SLR of economic burden estimations | UK | Medline, PubMed, the Cochrane library, NHS, eBMJ, the British Heart Foundation and references | NA | UK articles about economic burden of  congestive HF | NA | NA | NA |  |
| Stewart S et al. [35] | Resource use | 2002 | Economic burden estimation | UK | Estimates obtained from Scottish data and applied on an age and sex-specific basis to official mid-year population estimates for the UK | Patients with symptomatic left-ventricular systolic dysfunction and preserved left-ventricular systolic dysfunction in the UK | NA | NA | NHS | Prevalence-based |  |
| CIBIS-II Investigators and Health Economics Group [36] | Resource use | 2001 | Cost-of-illness study | Germany  UK  France | CIBIS II study.  Costs of drug therapy extracted from Vidal (France), Rote Liste (Germany), British National Formulary (UK) | HF (n=2,647) | Bisoprolol (n=1,327) | Placebo (n=1,320) | France, Germany: third party payer  UK: NHS | Prospective |  |
| Ekman M et al. [37] | Resource use  HF costs  Cost-effectiveness | 2001 | Cost-effectiveness analysis | Sweden | CIBIS-II study.  LIF and Apoteket AB. | Patients with symptomatic congestive HF class III or IV of NYHA class | Bisoprolol | Placebo | Societal | Prospective |  |
| Blue L et al. [38] | Resource use | 2001 | Clinical trial | UK | Hospital records department, Scottish NHS and the Registrar General's Office of Scotland | HF patients due to left ventricular systolic dysfunction (n=165) | Specialist nurse intervention (n=81) | UC (n=75) | NA | Randomized controlled trial |  |
| McMurray JJ, Stewart S. [39] | HF costs | 2000 | Review | UK  The Netherlands  Sweden  France | Previous publications | HF patients | NA | NA | NA | NA |  |
| Sculpher MJ et al. [40] | Resource use | 2000 | Cost-effectiveness analysis | UK | ATLAS trial | HF (n=3164 patients) | HD of lisinopril (n=1568) | LD of lisinopril (n=1596) | Health service | Randomized  controlled trial |  |
| Gonzalez-Loyola FE et al. [41] | Resource use  HF costs | 2022 | Economic burden estimation | Spain | SIDIAP database | Patients registered as presenting a new HF diagnosis (n=64,441) | After diagnosis | Before diagnosis | Healthcare system | Observational, retrospective cohort |  |
| Checa C et al. [42] | HF costs | 2022 | SLR including cost-of-illness studies | Spain (data from Sweden, the Netherlands and Ireland) | MEDLINE, CINAHL, Embase, Clinical Trials, WHO, Registry of International Clinical Trials, and Central Cochrane | Patients with advanced HF, III/IV NYHA classification, stage D of the ACCF/AHA, or under palliative care | Nurse-led case management model.  Community interventions including those commencing in hospital. | UC  Another nurse-led case management program. | NA | Articles including prospective and cohort studies. |  |
| Roalfe AK et al. [43] | Resource use | 2021 | Review of two studies | UK  The Netherlands | REFER and UHFO studies | Patients suspected of new onset HF in PC | NA | NA | NA | NA |  |
| Raat W et al. [44] | HF costs | 2020 | SLR of trials | Belgium | PubMed, Embase, and CENTRAL | Patients with a HF diagnosis | Multidisciplinary HF DMP | UC | NA | Randomized controlled trial |  |
| Rachamin Y et al. [45] | Resource use | 2020 | Epidemiology and treatment study | Switzerland | Swiss PC data from the FIRE project | Patients with an explicit HF diagnosis  (n=1,288) | NA | NA | NA | Retrospective, cross-sectional |  |
| Conangla L et al. [46] | Resource use | 2020 | Diagnostic accuracy study | Spain | NA | Ambulatory patients suspected of a new onset non-acute HF (n=162) | Lung Ultrasound | NA | NA | Prospective |  |
| Grustam AS et al. [47] | HF costs  Cost-effectiveness | 2018 | Cost-effectiveness analysis | The Netherlands | Previous publications | HF patients with 70 years or more (n=1,000) | HTM  NTS | UC | Third-party payer | Markov model approach |  |
| González-Guerrero JL et al. [48] | Resource use  HF costs  Cost-effectiveness | 2018 | Cost-effectiveness analysis | Spain | Regional Ministry of Health to the  Health Area of Caceres, Spanish NHS, Pricewa-  terhouseCoopers Consulting, EuroQol | Consecutive patients diagnosed  with acute HF (n=117) | DMP (n=59) | UC (n=58) | Societal and health-care system | Randomized and single-blind clinical trial |  |
| Taylor CJ et al. [49] | Resource use | 2017 | Diagnostic accuracy study | UK | REFER study | PC patients presenting HF symptoms | NA | NA | NA | Prospective, observational, diagnostic validation study |  |
| Comín-Colet J et al. [50] | Resource use  HF costs | 2016 | Clinical trial | Spain | iCOR trial | Patients with clinical diagnosis of HF (n=188) | UC including telemedicine | UC | Healthcare system | Single-center, randomized, open-label study |  |
| Sahlen KG et al. [51] | Resource use  HF costs  Cost-effectiveness | 2016 | Cost-effectiveness study | Sweden | PREFER trial and Statistics Sweden | Patients with a diagnosis of HF with NYHA class III-IV symptoms (n=72) | Palliative Advanced Home Care and HF Care, PREFER (n=36) | UC (n=36) | Healthcare system | Randomized controlled trial |  |
| Brännström M, Boman K. [52] | Resource use | 2014 | Clinical trial | Sweden | PREFER trial | Patients with a diagnosis of HF with NYHA class III-IV symptoms (n=72) | Palliative Advanced Home Care and HF Care, PREFER (n=36) | UC (n=36) | NA | Prospective randomized study with an open non-blinded design |  |
| Boyne JJ et al. [53] | Resource use | 2012 | Clinical trial | The Netherlands | TEHAF study | Consecutive HF patients in NYHA (n=382) | Telemonitoring  (n=197) | Nurse-led UC (n=185) | NA | Prospective open label, multicenter, randomized controlled study |  |
| Hancock HC et al. [54] | Resource use | 2012 | Pilot trial | UK | HF in Older People In Care Homes Trial | Randomized residents from care facilities with left ventricular systolic dysfunction (n=30) | Onsite HF service  (n=14) | UC (n=11) | NA | Pilot randomized controlled trial using a PROBE design |  |
| Lyngå P et al. [55] | Resource use | 2012 | Clinical trial | Sweden | WISH Trial | Patients hospitalized for HF with NYHA class III–IV (n=319) | Electronic  scales for daily transmission of body weight to the HF clinic (n=153) | Telephone contact by patients to the HF clinic in case of weight gain (n=166) | NA | Multicenter, randomized controlled trial |  |
| Kelder JC et al. [56] | Resource use | 2011 | Diagnostic accuracy study | The Netherlands | NA | Patients presenting  with symptoms and signs suggestive of HF (n=721) | NA | NA | NA | Cross-sectional study with external validation |  |
| Postmus D et al. [57] | Resource use  HF costs | 2011 | Clinical trial with economic evaluation | The Netherlands | COACH study | HF patients (n=1,023) | BS (n=340)  IS (n=344) | UC (n=339) | Health service | Multicenter, randomized controlled trial |  |
| De la Porte PW et al. [58] | Resource use  HF costs | 2007 | Clinical trial with economic evaluation | The Netherlands | Deventer–Alkmaar HF study | Patients with NYHA class III-IV HF (n=240) | Intensive  follow‐up (n=118) | UC (n=122) | Health service | Parallel group, randomized controlled trial |  |
| del Sindaco D et al. [59] | Resource use  HF costs | 2007 | Clinical trial with economic evaluation | Italy | NA | HF patients class III–IV discharged home after a hospitalization (n=173) | DMPs (n=86) | UC (n=87) | Health service | Randomized open trial |  |
| Agvall B et al. [60] | Resource use  HF costs | 2005 | Cost-of-illness study | Sweden | Data from PHC centers and hospital, Ödeshög study, Swedish National Medical Agency | Patients with a diagnosis of HF (n=115) | NA | NA | PHC | Retrospective |  |
| Atienza F et al. [61] | Resource use  HF costs | 2004 | Clinical trial with economic evaluation | Spain | Hospital Accounting Departments | HF patients 65 years or older (n=338) | Hospital discharge planning and close follow-up at a HF clinic (n=164) | UC (n=174) | Health service | Multicenter randomized trial |  |
| Ledwidge M et al. [62] | Resource use  HF costs | 2003 | Cost-of-illness study | Ireland | National Centre for Pharmacoeconomics in Ireland | Patients with a diagnosis of HF (n=98) | MC (n=51) | RC (n=47) | Healthcare provider | Prospective, randomized, controlled study |  |

ACCF/AHA: American College of Cardiology Foundation/American Heart Association; BS: Basic Support; CRD: Centre for Reviews and Dissemination; CV: Cardiovascular; DMP: Disease Management Program; EED: Economics Evaluation Database; ESC: European Society of Cardiology; HD: High Dose; HF: Heart Failure; HFpEF: Heart Failure Preserved Ejection Fraction; HFrEF: Heart Failure Reduced Ejection Fraction; HTM: Home Telemonitoring; IS: Intensive Support; LD: Low Dose; MC: Multidisciplinary Care; NA: Not Applicable; NHS: National Health System; NTS: Nurse Telephone Support; NYHA: New York Heart Association; PC: Primary Care; PHC: Primary Healthcare; RC: Routine Care; SLR: Systematic Literature Review; ST: Standard Therapy; UC: Usual Care; UK: United Kingdom; USA: United States of America

**Supplementary Table S2:** Studies reporting resource use associated with HF treatment

| **Reference** | | **Valuation year** | **Follow-up** | **Intervention vs Comparator** | **Resource use** | **Value (intervention)** | | | **Value (comparator)** | | **Units** | | | |  |  |
| --- | --- | --- | --- | --- | --- | --- | --- | --- | --- | --- | --- | --- | --- | --- | --- | --- |
| Jaarsma T et al. [14] | | 1999 | 1 year | NA | Hospitalization  Treatment regimens:   None   Diuretic monotherapy  ACEI monotherapy   Beta-blocker monotherapy   Diuretic and ACEI   Diuretic and beta-blocker   Diuretic and digoxin   ACEI and beta-blocker   Diuretic, ACEI and beta-blocker   Diuretic, ACEI and digoxin   Diuretic, ACEI and spironolactone   Diuretic, ACEI, beta-blocker and digoxin   Diuretic, ACEI, digoxin and spironolactone   Other | 24,868  100  7.0  8.6  5.3  9.6  15.1  4.8  3.6  7.0  7.5  9.8  4.2  3.3  2.7  11.5 | | | NA | | Admissions  % patients  % patients  % patients  % patients  % patients  % patients  % patients  % patients  % patients  % patients  % patients  % patients  % patients  % patients  % patients | | | |  |  |
| McEwan P et al. [16] | | 2019 | 18.2 months (mean) | Dapagliflozin  added to ST vs ST | HF medication   Diuretic   ACEI  ARB   Sacubitril/valsartan   Beta-blocker   Mineralocorticoid receptor antagonist   Digitalis  Hospitalization HF  Urgent HF visit | 93.4  56.1  28.4  10.5  96.0  71.5  18.8  820  32 | | | 93.5  56.1  26.7  10.9  96.2  70.6  18.6  925  54 | | % patients  % patients  % patients  % patients  % patients  % patients  % patients  Admissions per 1000 patients  Admissions per 1000 patients | | | |  |  |
| Escobar C et al. [17] | | 2015-2019 (cumulative values) | 5 years | NA | HF medication   Renin angiotensin system inhibitor   Angiotensin-converting enzyme inhibitor   ARB   Beta-blocker   Loop-diuretic   Aldosterone antagonist   Sacubitril/valsartan   Digoxin  PC visits  Laboratory requests  Radiology and other tests  Specialized visits  ER visits  Hospitalization for HF | 17,163 (100)  11,425 (66.6)  5,340 (31.1)  6,085 (35.5)  11,727 (68.3)  12,061 (70.3)  5,152 (30.0)  1,464 (8.5)  1,197 (7.0)  49.7  3.5  3.5  6.8  3.2  37.6 | | | NA | | Patients (%)  Patients (%)  Patients (%)  Patients (%)  Patients (%)  Patients (%)  Patients (%)  Patients (%)  Patients (%)  Per patient  Per patient  Per patient  Per patient  Per patient  Days | | | |  |  |
| Bundgaard JS et al. [18] | | 1998-2016 | NA | HF patients vs Control patients | Total patients  Medications   Antithrombotic and anticoagulants   Antihypertensive   Nitrates   Digoxin   Statin | 176,067  105,342 (60)  133,111 (76)  37,042 (21)  25,362 (14)  54,609 (31) | | | 176,067  58,897 (34)  81,610 (46)  13,290 (8)  7,941 (5)  32,162 (18) | | Number  Patients (%)  Patients (%)  Patients (%)  Patients (%)  Patients (%) | | | |  |  |
| Stafylas P et al. [20] | | 2009-2011 | 95.2 days (mean) | NA | Total patients   Annual hospitalization rate   Time till the first hospitalization   Hospitalizations, cumulative    1 month    6 months    12 months   Rehospitalizations  Patients during index hospitalization   Mean   Median   Mean time in ICU | 307  101 (32.9)  122.18  13 (4.2)  76 (24.8)  101 (32.9)  0.67  177  8.36  7.00  0.96 | | | NA | | Number  Admissions (%)  Days  Admissions (%)  Admissions (%)  Admissions (%)  Per patient  Number  Per patient  Per patient  Days per patient | | | |  |  |
| Corrao G et al. [24] | | 2011 | NA | Previous HF vs No previous HF | Total patients  Use of CV drugs   ACEI   ARBs   Beta-blockers   Diuretics   Aldosterone antagonists   Digital glycosides | 8,154  6,593 (80)  4,108 (50)  6,233 (76)  7,701 (94)  4,998 (61)  2,813 (35) | |  | 18,795  11,511 (61)  7,402 (39)  9,900 (53)  12,031 (64)  3,570 (19)  2,680 (14) | | Number  Patients (%)  Patients (%)  Patients (%)  Patients (%)  Patients (%)  Patients (%) | | | |  |  |
| Czech M et al. [26] | | 2013 | 1 year | NA | Hospitalization for HF  Hospitalization for other reasons  Outpatient appointments (scheduled)  Outpatient appointments (emergency)  Home visits (scheduled)  Home visits (emergency, due to intensification of HF symptoms)  Home visits (emergency, for other reasons)  Treatment in an ICU  Treatment in an ICU among patients requiring intensive care  Procedures   Coronarography   Balloon angioplasty   Stent implantation   CABG   Valve procedure   Complete blood count   ASPAT   Serum bilirubin   Serum glucose   Serum sodium   Serum potassium   Serum creatinine   Serum uric acid   Serum urea   Proteinogram or serum albumin level   Lipid profile   TSH and/or fT4   B-type natriuretic peptide   General urine test   Troponin   CPK and/or CPK-MB   International normalized ratio   ECG   Chest X-ray   Echocardiography   Spirometry   Holter ECG   Exercise test   Abdominal ultrasonography | 0.68  0.33  8.90  1.12  0.55  0.35  0.21  1.61  4.67  0.0608  0.0211  0.0251  0.0048  0.0016  1.63  1.1  0.69  6.36  1.72  1.84  1.56  0.58  1.08  0.27  1.44  0.47  0.07  1.67  0.4  0.4  0.49  4.12  0.89  0.89  0.19  0.36  0.24  0.02 | | | NA | | Per patient per year  Per patient per year  Per patient per year  Per patient per year  Per patient per year  Per patient per year  Per patient per year  Days per patient per visit  Days per patient per visit  Per patient per year  Per patient per year  Per patient per year  Per patient per year  Per patient per year  Per patient per year  Per patient per year  Per patient per year  Per patient per year  Per patient per year  Per patient per year  Per patient per year  Per patient per year  Per patient per year  Per patient per year  Per patient per year  Per patient per year  Per patient per year  Per patient per year  Per patient per year  Per patient per year  Per patient per year  Per patient per year  Per patient per year  Per patient per year  Per patient per year  Per patient per year  Per patient per year  Per patient per year | | | |  |  |
| Berger R et al. [27] | | 2003-2004 | 18 months maximum | Intensive patient management vs Nurse-Led MC and UC | Total patients  Prescriptions at discharge   ACEIs or ARBs   Beta-blockers   Spironolactone   Triple therapy   Combination of ACEI and ARB   Furosemide  Prescriptions at follow-up   ACEIs or ARBs   Beta-blockers   Spironolactone   Triple therapy   Combination of ACEI and ARB   Furosemide  Ambulatory visits at the HF specialist   Scheduled visits   Unscheduled visits  Telephone contacts with the HF specialist  Days in hospital due to HF | Intensive  92  91  82  45  7  0  85  92  88  53  37  44  76  564  336  228  263  488 | | | MC  96  88  76  42  4  0  78  88  92  49  20  39  72  511  220  291  103  1,254 | UC  90  89  73  37  7  0  81  87  76  46  9  0  79  229  229  1,588 | Number  Patients  Patients  Patients  Patients  Patients  Patients  Patients  Patients  Patients  Patients  Patients  Patients  Admissions  Admissions  Admissions  Admissions  Days | | | | | |
| Claes N et al. [29] | | 2001 | 1 year | NA | HF Hospitalizations   Planned   Referred by the GP   Internally referred or unknown   ER visit   Ambulance transport  Total in-hospital stay  Mean in-hospital stay  ICU visits  Mean ICU length of stay  Readmissions for HF or other CVDs  Mean time since last discharge | 19,398 (100)  4,209 (21.7)  8,926 (46.0)  621 (3.2)  14,568 (75.1)  3,788 (19.5)  286,938  14.8  5,580 (28.8)  3.8  50.9  3.2 | | | NA | | Admissions (%)  Admissions (%)  Admissions (%)  Admissions (%)  Admissions (%)  Admissions (%)  Days  Days  Admissions (%)  Days  % of patients  Months | | | |  |  |
| Jaarsma T et al. [30] | | 2002-2005 | 18 months | BS and IS vs UC | Total patients  Death or hospitalization because of HF  Time lost (mean)  Time lost (median)  Patients hospitalized   All causes   CVD   HF  Number of hospitalizations   All causes   CVD   HF  Duration of HF hospitalization (median)  Medication   ACEI or ARB   Beta-blockers   Diuretics   Digoxin   Calcium antagonists   Nitrates   Statins | BS  340  138 (41)  33,731  9,0  192 (57)  143 (42)  84 (25)  377  236  121  8.0  85  70  97  32  17  30  42 | | IS  344  132 (38)  34,268  7,5  194 (56)  147 (43)  92 (27)  408  255  134  9.5  81  63  95  29  13  33  35 | UC  339  141 (42)  39,960  12,0  181 (53)  143 (42)  84 (25)  375  255  120  12.0  82  65  96  30  18  32  37 | | Number  Patients (%)  Days  Days  Number (%)  Number (%)  Number (%)  Admissions  Admissions  Admissions  Days  % of patients  % of patients  % of patients  % of patients  % of patients  % of patients  % of patients | | | | |  |
| McMurray JJ et al. [32] | | 2003 | 41 months (median) | Candesartan added to conventional treatment vs Placebo i.e. conventional treatment for HF | Total patients   Total follow-up duration   Deaths   Admissions   Hospital days   Hospital days/admission   Admissions/patient   Hospital days/patient   Hospital days/patient-years  Hospitalized patients   Admissions/patient   Hospital days/patient  Hospitalization for worsening HF  CV procedures   Cardiac catheterization, as angiography   CABG   PTCA with stent   PTCA without stent   Implantation of cardioverter defibrillator   Implantation of pacemaker   Heart transplantation   Ventricular assistance device   Other cardiac surgery for HF   Other CV procedure/operation  Patients with CV procedure | HFrEF  2,289  6,504  642  4,181  36,981  17.67  3.63  32.05  11.37  1,462  5.71  50.43  1,052  1,009  311  60  66  20  81  129  24  7  11  300  538 (23.5) | HFpEF  1,514  4 434  237  2,510  22,942  9.14  1.66  15.15  5.17  912  2.75  25.16  402  681  262  46  78  15  9  67  0  1  11  192  342 (22.6) | | HFrEF  2,287  6,303  708  4,634  40,977  17.79  4.01  35.56  13.00  1,501  6.11  54.31  1,444  1,134  388  48  86  30  91  125  23  9  9  325  678 (29.6) | HFpEF  1,509  4,387  244  2,548  22,705  8.91  1.69  15.05  5.18  922  2.76  24.63  566  668  261  42  73  23  9  54  0  0  10  196  350 (23.2) | Number  Patient-years  Patients  Patients  Days  Days per admission  Admissions per patient  Days per patient  Days per patient-years  Number  Admissions per patient  Days per patient  Admissions  Number  Number  Number  Number  Number  Number  Number  Number  Number  Number  Number  Number (%) | | | |  |  |
| Scalvini S et al. [33] | | NA | 1 year | Home-based  Telecardiology vs UC | Total patients   Hospitalization   Patients with instability   Deaths | 231 (100)  56 (24)  60 (26)  6 (7) | | | 180 (100)  61 (34)  74 (41)  22 (12) | | Number (%)  Admission (%)  Number (%)  Patients (%) | | | |  |  |
| Laceya L, Tabbererb M [34] | | 1995 | 1 year | NA | Total chronic HF patients in UK   GP visits   Drug prescriptions   Hospital admissions due to HF   Outpatient visits | 650,000  7.6 million  5.3 million  86,101  537,945 | | | NA | | Number  Number  Number  Number  Number | | | |  |  |
| Stewart S et al. [35] | | 1995 | 1 year | NA | Total patients requiring HF treatment  GP consultations related to HF  Outpatient visits referring to GP  Total prescriptions for chronic HF   Diuretics   ACEI   ASA   Cardiac glycosides   Nitrates   Calcium antagonists  Primary admissions  Post-discharge outpatient visits  Secondary admissions | 988,000  2.4 million  652,000  21 million  6.64 (96)  4.92 (59)  3.05 (44)  2.42 (35)  2.35 (34)  1.58 (23)  135,000  299,000  176,000 | | | NA | | Number  Number  Number  Number  Patients (%)  Patients (%)  Patients (%)  Patients (%)  Patients (%)  Patients (%)  Number  Number  Number | | | |  |  |
| CIBIS-II Investigators and Health Economics Group [36] | | 1997-1998 | 1.3 years | Bisoprolol vs Placebo | Total patients   Hospital admissions   Time in hospital per admission   Hospital admissions per patient   Time in hospital per patient  Patients hospitalized   Admissions per patient hospitalized   Time in hospital per patient hospitalized  Patients with worsening HF  Hospital admissions by worsening HF  Time of bed occupancy   Coronary ICU   Cardiology ward   General Medical ward   Other | 1,327  739  13.7  0.6  7.6  454 (34)  1.6  22.2  159 (12.0)  211  10,085  796  4,379  2,787  2,123 | | | 1,320  1,013  12.1  0.8  9.3  530 (40)  1.9  23.1  232 (17.6)  393  12,144  1,056  5,280  3,300  2,508 | | Number  Number  Days  Number  Days  Number (%)  Number  Days  Number (%)  Number  Days  Days  Days  Days  Days | | | |  |  |
| Ekman M et al. [37] | | 1999 | 1.3 years (mean)  2.3 years (max) | Bisoprolol vs Placebo | HF worsening  Ventricular tachycardia/fibrillation  Arrhythmia  Bradycardia  Hypotension  Stroke  Myocardial infarction  Angina  Cardiogenic shock  Cardiac transplant surgery  Revascularization  Other cardiac surgery  Other CV  Non-CV  Total hospitalizations | 0.159  0.005  0.020  0.011  0.003  0.023  0.012  0.035  0.005  0.005  0.009  0.001  0.063  0.150  0.500 | | | 0.298  0.019  0.033  0.002  0.008  0.013  0.008  0.041  0.005  0.004  0.010  0.001  0.061  0.195  0.698 | | Per patient  Per patient  Per patient  Per patient  Per patient  Per patient  Per patient  Per patient  Per patient  Per patient  Per patient  Per patient  Per patient  Per patient  Per patient | | | |  |  |
| Blue L et al. [38] | | 1997-1999 | 12 months (mean) | Specialist nurse intervention vs UC | Total patients  Medication   Loop diuretic   Thiazide diuretic   ACEI   Digoxin   Beta-blocker   Oral or transdermal nitrate   CCB  Patients readmitted   All causes   Worsening HF  Readmissions (No/patient/month)   All causes   Worsening HF  Mean time in hospital   All causes   Worsening HF | 81 (100)  76 (94)  5 (6)  65 (80)  35 (43)  2 (2)  21 (26)  8 (10)  47 (56)  12 (14)  86 (0.124)  19 (0.027)  10.3  3.43 | | | 74 (100)  68 (92)  4 (5)  53 (72)  31 (42)  5 (7)  26 (35)  17 (23)  49 (60)  26 (32)  114 (0.174)  45 (0.069)  16.7  7.46 | | Number (%)  Patients (%)  Patients (%)  Patients (%)  Patients (%)  Patients (%)  Patients (%)  Patients (%)  Number (%)  Number (%)  Number (per patient per month)  Number (per patient per month)  Days  Days | | | |  |  |
| Sculpher MJ et al. [40] | | 1997-1998 | 46 months (median) | HD lisinopril vs LD lisinopril | Total patients  Cardiology   HF   Other cardiology  Other medical  General surgery  Thoracic medicine  Orthopedics  Cardiothoracic  Other  Total  Total in-patient time in hospital  Day-case hospital visits | 1,568  9.5  6.0  3.5  2.3  2.3  1.1  0.6  0.5  2.2  18.5  28,941  600 | | | 1,596  12.2  8.1  4.1  3.0  2.5  1.5  0.7  0.3  2.4  22.5  35,906  698 | | Number  Days per patient  Days per patient  Days per patient  Days per patient  Days per patient  Days per patient  Days per patient  Days per patient  Days per patient  Days per patient  Days  Number | | | |  |  |
| Gonzalez-Loyola FE et al. [41] | | 2010-2013 | 4 years | After diagnosis vs Before diagnosis | Nurse at PHC  Nurse at patient's home  Laboratory test  GP at PHC  GP at patient's home  PC emergency visits  Total | 1,222,671  318,662  164,861  1,320,889  128,248  30,640  3,185,971 | |  | 1,198,277  181,826  180,531  1,526,364  86,240  8,406  3,181,644 | | Number  Number  Number  Number  Number  Number  Number | | | |  |  |
| Rachamin Y et al. [45] | | 2016-2019 | 12 months | NA | Medication   Renin–angiotensin–aldosterone system inhibitors   Beta-blockers   CCBs   Diuretics   Cardiac therapy   Others    with peripheral vasodilators    with other antihypertensives  Monitoring   Median consultations   Measurements    with potassium measurement    with creatinine measurement    with BNP variant measurement    with blood pressure measurement    with heart rate measurement    with weight measurement  Prescriptions for cardiac therapy   ACEIs    Lisinopril    Perindopril    Ramipril    Enalapril    Others   ARBs    Valsartan    Candesartan    Losartan    Irbesartan    Olmesartan medoxomil    Others   ARNIs    Sacubitril/ valsartan    Glyceryl trinitrate    Amiodarone    Digoxin    Isosorbide dinitrate    Nicorandil    Others | 73.6  67.8  34.6  86.1  40.1  0.8  3.0  17  47.1  74.6  7.6  76.4  72.5  51.0  850 (100)  472 (56)  160 (19)  156 (18)  51 (6)  11 (1)  608 (100)  228 (38)  212 (35)  74 (12)  41 (7)  33 (5)  20 (3)  746 (100)  77 (10)  204 (27)  187 (25)  115 (15)  82 (11)  44 (6)  114 (15) | | | NA | | % patients  % patients  % patients  % patients  % patients  % patients  % patients  Number per year  % patients  % patients  % patients  % patients  % patients  % patients  Patients (%)  Patients (%)  Patients (%)  Patients (%)  Patients (%)  Patients (%)  Patients (%)  Patients (%)  Patients (%)  Patients (%)  Patients (%)  Patients (%)  Patients (%)  Patients (%)  Patients (%)  Patients (%)  Patients (%)  Patients (%)  Patients (%)  Patients (%)  Patients (%) | | | |  |  |
| Conangla L et al. [46] | | 2015-2018 | NA | HF patients vs Patients without HF | Total patients   ACEI or ARB   Beta-blockers   MRA   Loop diuretic   Thiazide   Digoxin   Calcium antagonists   Anticoagulants   ASA | 33  25 (75.8)  14 (42.4)  1 (3.0)  5 (15.2)  9 (27.3)  2 (6.1)  24 (72.7)  12 (36.4)  7 (21.2) | |  | 129  74 (57.4)  25 (19.4)  0 (0.0)  10 (7.8)  43 (33.3)  3 (2.3)  19 (14.7)  8 (6.2)  37 (28.7) | | Number  Patients (%)  Patients (%)  Patients (%)  Patients (%)  Patients (%)  Patients (%)  Patients (%)  Patients (%)  Patients (%) | | | |  |  |
| González-Guerrero JL et al. [48] | 2010 | | 1 year | DMP VS UC | Patients  Hospitalization (days)  Specialist office visits   New patient   Follow-up  Telephone contacts   Physician   Nurse  PC practice visits   Office   Home  Emergency visits  Rehabilitation  Residential care | 59  437  7  176  102  58  891  60  35  54  612 | | | 58  576  8  39  -  -  1026  100  48  -  452 | | Number  Days  Number  Number  Number  Number  Number  Number  Number  Sessions  Days | | | |  |  |
| Taylor CJ et al. [49] | 2011-2013 | | NA | HF patients vs Patients without HF | Total patients   ACEIs   Beta-blockers   ARBs   Diuretics | 104  38 (36.5)  46 (44.2)  19 (18.3)  61 (58.6) | |  | 200  60 (30.0)  36 (18.0)  39 (19.5)  75 (37.5) | | Number  Patients (%)  Patients (%)  Patients (%)  Patients (%) | |  |  |  |  |
| Comín-Colet J et al. [50] | | 2010-2012 | 6 months | Telemedicine+ UC vs UC | Patients  Treatment   ACEI or ARBs   Beta-blockers   Aldosterone antagonists   Digoxin   Loop diuretics   Hydralazine-nitrate combination   Antiplatelet therapy/anticoagulant  HF hospitalization   Total events   Patients with events   Incidence   Rate   Readmissions   Time in hospital   Mean hospital stay | 81  49 (61)  67 (83)  23 (28)  9 (11)  81 (100)  21 (26)  71 (88)  15  11  30.4  11 (13.6)  0.2  2.2  12.3 | | | 97  59 (61)  82 (84)  24 (25)  14 (14)  93 (96)  27 (28)  81 (84)  40  32  81.6  32 (33.0)  0.4  6.4  16.2 | | Number  Patients (%)  Patients (%)  Patients (%)  Patients (%)  Patients (%)  Patients (%)  Patients (%)  Number  Number  Per 100 patient-years  Patients (%)  Per patient  Days  Days per admission | | | |  |  |
| Sahlen KG et al. [51] | | 2011-2013 | 6 months | PREFER vs UC | Total patients  GP  Other medical professionals  Emergency transport  Hospital care | 36  295  2,381  11  103 | | | 36  144  238  47  309 | | Number  Hours  Hours  Use  Days | | | |  |  |
| Brännström M, Boman K. [52] | | 2011-2012 | 6 months | PREFER vs UC | Total patients  Hospitalizations  Hospital outpatient clinic   Physician visits   Physician (phone calls, prescriptions)   Nurse visits   Nurse (phone calls, prescriptions)  PHC Centers   Physician visits, PHC   Physician (phone calls, prescriptions)   Nurse visits, PHC   Nurse (phone calls, prescriptions)   Physician visits, home   Nurse visits, home | 36  15 (0.42)  27 (1)  42 (3)  4 (1)  8, (1)  9 (1)  30 (1)  29 (1)  59 (3)  0 (0)  11 (2) | | | 36  53 (1.47)  133 (3)  86 (3)  60 (2)  44 (2)  54 (2)  145 (1)  61 (2)  153 (4)  14 (2)  109 (5) | | Number  Number (mean)  Number (median)  Number (median)  Number (median)  Number (median)  Number (median)  Number (median)  Number (median)  Number (median)  Number (median)  Number (median) | | | |  |  |
| Boyne JJ et al. [53] | | 2007-2009 | 1 year | Telemonitoring vs Nurse-led UC | Total patients  HF patients admissions   One admission   Two admissions   >2 admissions  All admissions for HF  Total time hospitalization by HF | 197  18 (9.1)  13 (6.5)  4 (2.0)  1 (0.5)  24  253 | | | 185  25 (13.5)  14 (7.6)  7 (3.8)  4 (2.2)  43  330 | | Number  Number (%)  Number (%)  Number (%)  Number (%)  Number  Days | | | |  |  |
| Hancock HC et al. [54] | | 2009-2010 | 14 months | Onsite HF service vs UC | Results for 6 months   Total patients   Drug treatment    ACEI + Beta-blocker    ACEI    Ramipril    Beta-blocker    Bisoprolol    Spironolactone   HF hospitalization  Results for 12 months   Total patients   Drug treatment    ACEI + Beta-blocker    ACEI    Ramipril    Beta-blocker    Bisoprolol    Spironolactone    HF hospitalization | 14  10 (71)  13 (93)  11 (79)  12 (86)  11 (79)  2 (14)  0 (0)  13  7 (54)  11 (85)  10 (77)  7 (54)  7 (54)  2 (15)  0 (0) | | | 14  5 (45)  5 (45)  4 (36)  7 (64)  5 (45)  0 (0)  0 (0)  8  5 (63)  6 (75)  5 (63)  5 (63)  5 (63)  0 (0)  0 (0) | | Number  Patients (%)  Patients (%)  Patients (%)  Patients (%)  Patients (%)  Patients (%)  Number (%)  Number  Patients (%)  Patients (%)  Patients (%)  Patients (%)  Patients (%)  Patients (%)  Number (%) | | | |  |  |
| Lyngå P et al. [55] | | NA | 12 months | Electronic scales vs Telephone contact | Total patients  Previous HF  Hospitalizations for HF in previous year  Cardiac hospitalization during follow-up  Medication:   ACEI   ARB   Beta-blocker   Aldosterone antagonist   Digoxin   Warfarin | 153  124 (74.7)  59 (35.5)  70 (42.2)  111 (66.9)  44 (26.5)  155 (93.4)  72 (43.4)  40 (24.1)  92 (55.4) | | | 166  117 (76.5)  71 (46.4)  70 (45.8)  101 (66.0)  51 (33.3)  140 (91.5)  64 (41.8)  29 (19.0)  93 (60.8) | | Number  Patients (%)  Patients (%)  Patients (%)  Patients (%)  Patients (%)  Patients (%)  Patients (%)  Patients (%)  Patients (%) | | | |  |  |
| Kelder JC et al. [56] | | NA | 6 months | HF patients vs Patients without HF | Total patients   Loop diuretic   ACEI or ARB II   Digoxin   Nonsteroidal anti-inflammatory drug | 207  114 (55.1)  76 (36.7)  23 (11.1)  17 (8.2) | |  | 514  119 (23.2)  89 (17.3)  17 (3.3)  32 (6.2) | | Number  Patients (%)  Patients (%)  Patients (%)  Patients (%) | | | |  |  |
| Postmus D et al. [57] | | 2009 | 18 months | BS and IS vs UC | Total patients  Intervention   Inpatient hospital visits   Home visits   Outpatient visits   Telephone contacts   Multidisciplinary advice  Hospitalization   CV related   Non-CV related   Short-stay admission   HF-related diagnostics | BS  340  0.41  0.03  6.16  3.55  0  7.58  4.39  0.18  1.10 | | IS  344  0.89  2.14  7.46  9.21  1.28  8.10  4.96  0.17  1.42 | UC  339  0.16  0  3.38  0.14  0  8.44  3.76  0.17  1.20 | | Number  Per patient  Per patient  Per patient  Per patient  Per patient  Days per patient  Days per patient  Days per patient  Days per patient | | | |  |  |
| De la Porte PW et al. [58] | | 2000-2003 | 12 months | Intensive follow-up vs UC | Total patients  Hospitalization for chronic HF and/or death  Death (all‐cause)  Time of hospitalization | 118  23 (20.7)  12 (10.8)  359 (324) | | | 122  47 (42.2)  23 (20.6)  644 (578) | | Number  Number (per 100 patient years)  Number (per 100 patient years)  Days (per 100 patient years) | | | |  |  |
| del Sindaco D et al. [59] | | 2001-2002 | 2 years | DMP vs UC | Total patients  Death or HF admission  HF admission  All-cause admission  All-cause death  CV death  Telephone calls (mean time)  Visits (mean time)  Nurse  Cardiologist | 86  40 (46.5)  28 (32.5)  48 (55.8)  27 (31.4)  21 (24.4)  731 (15)  427 (30)  397  304 | | | 87  56 (64.4)  49 (40.3)  65 (74.7)  32 (36.8)  25 (28.7) | | Number  Number (%)  Number (%)  Number (%)  Number (%)  Number (%)  Number (min)  Number (min)  Hours  Hours | | | |  |  |
| Agvall B et al. [60] | | 1999-2000 | NA | NA | Total patients  Ongoing medication   Beta-blockers   Diuretics   ACEI   ARB II   CCBs   Digoxin   Statins   Long-acting nitrates   ASA   Warfarin  Hospital care  Inpatient care   ICU   Hospital ward  Outpatient care   Visit to the doctor   Visit to nurse  PHC  GP   Visit to the doctor   Home visit   Telephone contact   Prescription of drugs  Nurse   Visit to nurse   Visit to asthma nurse   Visit to a diabetes nurse   Visit to hypertension nurse  District nurse   Visit to district nurse   Home visit  Paramedical staff   Occupational therapist   Physiotherapist   Chiropodist  Nursing home | 115  81 (70)  85 (74)  77 (67)  3 (3)  37 (32)  32 (28)  26 (23)  58 (50)  61 (53)  29 (25)  0.2  4.3  0.7  0.2  4.6  0.1  1.0  1.5  0.3  0.2  1.0  0.4  3.3  5.2  0.8  0.6  0.5  4.4 | | | NA | | Number  Patients (%)  Patients (%)  Patients (%)  Patients (%)  Patients (%)  Patients (%)  Patients (%)  Patients (%)  Patients (%)  Patients (%)  Days per visit  Days per visit  Per patient  Per patient  Per patient  Per patient  Per patient  Per patient  Per patient  Per patient  Per patient  Per patient  Per patient  Per patient  Per patient  Per patient  Per patient  Per patient | | | |  |  |
| Atienza F et al. [61] | | 1999-2000 | 509 days (mean) | Close follow-up vs UC | Total patients  Medication at hospital discharge   Diuretic   Spironolactone   ACEI   Digoxin   Nitrate   Beta-blocker   ARB II   Dihydropyridine   Amiodarone   Antiplatelet   Anticoagulant  Rate of events  Rate of readmitted patients   For HF   Not for HF  Rate of readmissions   For HF   Not for HF  Rate of deaths  Total time of hospitalization  HF time of hospitalization | 164  149 (91)  61 (37)  110 (67)  84 (51)  50 (30)  31 (19)  21 (13)  18 (11)  22 (13)  64 (39)  57 (35)  0.70  0.31  0.18  0.21  0.56  0.27  0.29  0.14  1,059  500 | | | 174  166 (95)  66 (38)  118 (68)  84 (48)  58 (33)  20 (12)  16 (10)  20 (12)  17 (10)  64 (37)  58 (33)  1.17  0.47  0.37  0.24  0.93  0.57  0.36  0.24  2,090  1,394 | | | Number  Patients (%)  Patients (%)  Patients (%)  Patients (%)  Patients (%)  Patients (%)  Patients (%)  Patients (%)  Patients (%)  Patients (%)  Patients (%)  Per year  Per year  Per year  Per year  Per year  Per year  Per year  Per year  Days  Days | |  |  |  |
| Ledwidge M et al. [62] | | 1998-2000 | 3 months | NA | Total patients  MDC specialist nurse service   Average visits per patient   Average time with patient   Average time with carer   Total specialist nurse visits   Total time   Average service time per patient  MDC specialist dietician service   Average visits per patient   Average time with patient   Average time with carer   Total specialist nurse visits   Total time   Average service time per patient | 51  3.6  84.8  18.9  185  5,290  4.2  1.7  60.0  16.9  85  3,920  2.2 | | | NA | | | Number  Number  Minutes  Minutes  Number  Minutes  Hours  Number  Minutes  Minutes  Number  Minutes  Hours | |  |  |  |

ACEI: ACE inhibitors; ARB: Angiotensin Receptor Blocker; ARNI: Angiotensin Receptor Neprilysin Inhibitor; ASA: Acetylsalicylic Acid; ASPAT: Asparagine-oxo-acid Transaminase; BNP: Brain Natriuretic Peptide; BS: Basic Support; CABG: Coronary Artery Bypass Graft; CCB: Calcium Channel Blocker; CPK: Creatine Phosphokinase; CV: Cardiovascular; CVD: Cardiovascular Disease; DMP: Disease Management Program; ECG: Electrocardiogram; ER: Emergency Room; GP: General Practitioner; HF: Heart Failure; HD: High Dose; ICU: Intensive Care Unit; IS: Intensive Support; LD: Low Dose; MC: Multidisciplinary Care; MRA: Magnetic Resonance Angiogram; NA: Not Applicable; NT-proBNP: N-terminal pro–B-type natriuretic peptide; PHC: Primary Healthcare; PTCA: Percutaneous Transluminal Coronary Angioplasty; RC: Routine Care; ST: Standard Therapy; TSH: Thyroid-Stimulating Hormone, Thyrotropin; UC: Usual Care; UK: United Kingdom

**Supplementary Table S3:** Studies reporting costs associated with HF treatment

| **Reference** | **Year of valuation** | **Follow-up** | **Intervention vs Comparator** | **Cost categories** | **Costs (intervention)** | | **Costs (comparator)** | | **Currency and units** | **Discount rate** |  |
| --- | --- | --- | --- | --- | --- | --- | --- | --- | --- | --- | --- |
| Informe de Posicionamiento Terapéutico de Dapagliflozina [15] | 2020 | 18.2 months (median) | Dapagliflozin vs Sacubitril-Valsartan and Enalapril and Valsartan and Placebo | Dapagliflozin  Sacubitril-Valsartan  Enalapril  Valsartan | 401.5  -  -  - | | -  1,689.95  25.55  273.75 | | € per patient per year  € per patient per year  € per patient per year  € per patient per year | NA |  |
| McEwan P et al. [16] | 2019 | 18.2 months (median) | Dapagliflozin  added to ST vs ST | **UK**  Treatment, monitoring, adverse events  Worsening HF events and CV death  Background resource use  **Germany**  Treatment, monitoring, adverse events  Worsening HF events and CV death  Background resource use  **Spain**  Treatment, monitoring, adverse events  Worsening HF events and CV death  Background resource use | **18,722**  4,892  4,394  9,436  **25,328**  7,637  9,944  7,747  **24,330**  10,139  5,425  8,766 | | **15,550**  2,187  4,825  8,537  **22,647**  5,059  10,598  6,990  **19,642**  5,785  5,945  7,912 | | **€ per patient per year**  € per patient per year  € per patient per year  € per patient per year  **€ per patient per year**  € per patient per year  € per patient per year  € per patient per year  **€ per patient per year**  € per patient per year  € per patient per year  € per patient per year | 3.5% in the UK  3% in Spain and Germany |  |
| Escobar C et al. [17] | 2015-2019 | 5 years | NA | **Cumulative cost for 5 years**  PC visits  Laboratory requests  Radiology and other tests  Specialized visits  ER visits  Hospitalization   HF   CKD   MI   Stroke   PAD  Total medication cost:   Diabetes medication   HF medication   CVD medication  Sanitary Cost (total direct costs)  Indirect Cost/Sick Leave | **15,373**  1,202  113  131  635  368  11,649  7,842  2,504  416  616  271  1,083  486  417  181  15,151  222 | | NA | | **€ per patient**  € per patient  € per patient  € per patient  € per patient  € per patient  € per patient  € per patient  € per patient  € per patient  € per patient  € per patient  € per patient  € per patient  € per patient  € per patient  € per patient  € per patient | NA |  |
| Bundgaard JS et al. [18] | 1998-2016 | NA | HF patients vs Control patients | **Total costs**  Healthcare (direct costs)   Outpatient services   Inpatient admissions   Prescription drugs   Primary health sector   Psychiatric outpatient services   Psychiatric inpatient admissions  Home care   Care   Practical help  Indirect costs, foregone earnings | **17,093**  11,926  2,050  7,844  1,249  578  33  172  2,442  2,040  402  2,726 | | NA | | **€ per patient per year**  € per patient per year  € per patient per year  € per patient per year  € per patient per year  € per patient per year  € per patient per year  € per patient per year  € per patient per year  € per patient per year  € per patient per year  € per patient per year | NA |  |
| Stafylas P et al. [20] | 2009-2011 | 95.22 days (mean) | NA | **Total cost**  Medications  Physicians  Laboratory  Hospitalizations | **4,410.96**  632.95  154.61  539.73  3,226.95 | |  | | **€ per patient**  € per patient  € per patient  € per patient  € per patient | NA |  |
| Murphy TM et al. [21] | 2013 | 1 year | HFrEF vs HFpEF | **All Costs**  Index Admission  Readmission Costs   ADHF   Death   Elective CV   Elective non-CV   Emergency CV   Emergency non-CV   Other  Outpatient Clinical Workload (0-3 months)   Unscheduled   Scheduled (including Titration)   Telephone (outbound)   Telephone (inbound)  Outpatient Clinical Workload (3-12 months)   Unscheduled   Scheduled (including Titration)   Telephone (outbound)   Telephone (inbound)  Medications  GP visits | **13,011**  7,660  4,287  1,150  116  134  216  584  2,039  47  791  83  617  77  15  751  73  643  20  14  596  594 | | **12,206**  6,302  5,396  1,150  73  32  79  655  3,386  21  693  80  528  71  14  593  78  485  18  11  527  569 | | **€ per patient**  € per patient  € per patient  € per patient  € per patient  € per patient  € per patient  € per patient  € per patient  € per patient  € per patient  € per patient  € per patient  € per patient  € per patient  € per patient  € per patient  € per patient  € per patient  € per patient  € per patient  € per patient | 5% | |
| Menafoglio A et al. [23] | 2011-2012 | NA | CV evaluation including ECG vs CV evaluation  using only medical history and physical examination | **Evaluation costs for overall program**  Preparticipation CV evaluation  Echocardiogram  Exercise stress test  24 h Holter monitoring  24 h blood pressure monitoring  Cardiac MRI  ECG with exposure to adenosine  ECG  Signal-averaged ECG  Genetic test for long QT syndrome  Electrophysiological study and radiofrequency ablation | **141,559**  92.4 (100)  280.56 (5.6)  141.10 (4.0)  175.62 (1.9)  78.93 (0.7)  633.88 (0.5)  140.02 (0.4)  24.23 (0.4)  62.26 (0.1)  3,107 (0.1)  8,716 (0.1) | | NA | | **€**  € (% participants)  € (% participants)  € (% participants)  € (% participants)  € (% participants)  € (% participants)  € (% participants)  € (% participants)  € (% participants)  € (% participants)  € (% participants) | NA |  |
| Corrao G et al. [24] | 2011 | 1 year | HF cohort vs Referent cohort | Patients  **Total**  **Without index hospitalization**  **With index hospitalization**  Inpatient cost sources   Without index hospitalization   With index hospitalization  Outpatient cost sources   Drugs   Visits, procedures, and lab tests | 26,949  **119,335 (6.8)**  **193,896 (11.1)**  103,211 (5.9)  177,772 (10.2)  8,823 (0.5)  7,301 (0.4) | | 26,949  **24,563 (1.4)**  16,767 (1.0)  3,624 (0.2)  4,171 (0.2) | | Number  **Thousand € (per capita)**  **Thousand € (per capita)**  Thousand € (per capita)  Thousand € (per capita)  Thousand € (per capita)  Thousand € (per capita) | NA |  |
| Delgado JF et al. [25] | 2010 | 1 year | NA | **Total cost**  Total healthcare costs   PC visits   Specialist visits   Medication   PC emergency services   Hospital emergency departments   Medical transport   Hospital admission  Total formal care costs   Formal home care   Day care centers   Telehealth   Nursing homes  Informal care costs | **12,995-18,220**  4,860.14  172.39  380.14  1,352.5  31.32  82.43  114.04  2,727.32  451.43-636.68  281.92-467.16  67  14.41  88.11  7,683.11-12,723 | | NA | | **€ per patient**  € per patient  € per patient  € per patient  € per patient  € per patient  € per patient  € per patient  € per patient  € per patient  € per patient  € per patient  € per patient  € per patient  € per patient | NA |  |
| Czech M et al. [26] | 2011 | 1 year | NA | **Cost of treatment**   Treatment in ICUs   Hospitalization for HF   Hospitalization for other reasons   Outpatient appointments   Home visits   Outpatient diagnostic procedures   Drugs  **Cost of HF patients’ hospitalization**   Treatment in ICUs   Hospitalization | **1,793.79**  778.83  637.02  233.14  84.11  16.26  4.18  40.25  **1,725.8**  1,137.37  588.43 | | NA | | **€ per patient per year**  € per patient per year  € per patient per year  € per patient per year  € per patient per year  € per patient per year  € per patient per year  € per patient per year  **€ per patient per year**  € per patient per year  € per patient per year | NA |  |
| Neumann T et al. [28] | 2006 | 1 year | NA | **All facilities**  Health protection  Outpatient facilities   Physician’s practices   Other medical practices   Pharmacies   Medical product suppliers/retailers   Outpatient departments   Other outpatient care facilities  Inpatient/day-patient facilities   Hospitals   Prevention/rehabilitation facilities   Inpatient/day-patient care  Emergency services  Administration  Other facilities and private households  Abroad | **2,879**  3  784  162  29  287  59  239  7  1,721  1,304  11  407  57  147  164  2 | | NA | | **Million € per year**  Million € per year  Million € per year  Million € per year  Million € per year  Million € per year  Million € per year  Million € per year  Million € per year  Million € per year  Million € per year  Million € per year  Million € per year  Million € per year  Million € per year  Million € per year  Million € per year | NA |  |
| Claes N et al. [29] | 2001 | 1 year | NA | **Total costs**  Hospitalization  Pharmaceutical  Medical care  Imaging  Clinical biology  Implants and prostheses  Others | **94,113,827 (100)**  63,421,845 (67.4)  5,588,825 (5.9)  13,763,049 (14.6)  8,832,802 (9.4)  1,563,441 (1.7)  200,440 (0.2)  743,425 (0.8) | | NA | | **€ per year (% total cost)**  € per year (% total cost)  € per year (% total cost)  € per year (% total cost)  € per year (% total cost)  € per year (% total cost)  € per year (% total cost)  € per year (% total cost) | NA |  |
| McMurray JJ et al. [32] | 2003 | 41 months | Candesartan added to conventional treatment vs Placebo i.e. conventional treatment for HF | **France: total costs**  Hospitalizations  CV procedures  Concomitant medication  Study drug   Worsening HF   MI   Unstable angina   Stroke   Transient ischemic attack   Cardiogenic shock   Atrial tachyarrhythmia   Ventricular arrhythmia   Pulmonary embolism   Other CV event   Cancer (neoplasm)   Other non-CV event   Cardiac catheterizations including angiography   CABG   PTCA with stent   PTCA without stent   Implantation of cardioverter defibrillator   Implantation of pacemaker   Heart transplantation   Ventricular assist device   Other cardiac surgery for HF   Other CV procedure/operation   ICU/CCU   Cardiology ward   General medical ward   Non-CV admission   Visit GP   Laboratory test—blood biochemistry   Candesartan 4 mg   Candesartan 8 mg   Candesartan 16 mg   Candesartan 32 mg  **Germany: total costs**  Hospitalizations  CV procedures  Concomitant medication  Study drug   Worsening HF   MI   Unstable angina   Stroke   Transient ischemic attack   Cardiogenic shock   Atrial tachyarrhythmia   Ventricular arrhythmia   Pulmonary embolism   Other CV event   Cancer (neoplasm)   Other non-CV event   Cardiac catheterizations including angiography   CABG   PTCA with stent   PTCA without stent   Implantation of cardioverter defibrillator   Implantation of pacemaker   Heart transplantation   Ventricular assist device   Other cardiac surgery for HF   Other CV procedure/operation   ICU/coronary care unit   Cardiology ward   General medical ward   Non-CV admission   Visit GP   Laboratory test—blood biochemistry   Candesartan 4 mg   Candesartan 8 mg   Candesartan 16 mg   Candesartan 32 mg  **UK: total costs**  Hospitalizations  CV procedures  Concomitant medication  Study drug   Worsening HF   MI   Unstable angina   Stroke    Haemorrhagic    Ischaemic/Unknown/Other   Transient ischemic attack   Cardiogenic shock   Atrial tachyarrhythmia   Ventricular arrhythmia   Pulmonary embolism   Other CV event   Cancer (neoplasm)   Other non-CV event   Cardiac catheterizations including angiography   CABG   PTCA with stent   PTCA without stent   Implantation of cardioverter defibrillator   Implantation of pacemaker   Heart transplantation   Ventricular assist device   Other cardiac surgery for HF   Other CV procedure/operation   ICU/coronary care unit   Cardiology ward   General medical ward   Non-CV admission   Visit GP   Laboratory test—blood biochemistry   Candesartan 4 mg   Candesartan 8 mg   Candesartan 16 mg   Candesartan 32 mg | HFrEF  **11.67**  5.63  3.39  3.50  0.90  4,174  4,579  2,526  4,394  2,520  2,779  2,675  2,804  4,969  3,257  4,826  4,560  3,199  15,289  6,166  2,981  8,557  8,581  73,983  7,294  18,303  7,426  1,611  682  520  629  18.60  9  0.62  0.81  0.88  1.15  **9.39**  3.60  2.94  1.81  1.04  2 951  4 140  1 944  3 855  2 445  2 398  1 876  4 615  3 929  1 770  3 288  4 560  1 517  11 363  3 209  2 736  23 299  6 575  44 864  6 903  13 906  6 221  871  265  240  303  39.20  1.79  0.76  0.88  1.04  1.24  **6.44**  2.00  1.94  1.57  0.93  2,479  2,333  1,312  2,953  3,889  1,648  1,952  1,610  1,610  2,413  1,561  2,236  1,409  1,903  10,829  6,638  4,255  26,192  5,976  28,445  68,082  10,367  4,188  2,127  723  471  637  33.18  39.14  0.55  0.65  0.83  1.05 | HFpEF  **9.91**  4.84  2.61  1.53  0.93  4,174  4,579  2,526  4,394  2,520  2,779  2,675  2,804  4,969  3,257  4,826  4,560  3,199  15,289  6,166  2,981  8,557  8,581  73,983  7,294  18,303  7,426  1,611  682  520  629  18.60  9  0.62  0.81  0.88  1.15  **7.55**  2.92  1.93  1.63  1.07  2 951  4 140  1 944  3 855  2 445  2 398  1 876  4 615  3 929  1 770  3 288  4 560  1 517  11 363  3 209  2 736  23 299  6 575  44 864  6 903  13 906  6 221  871  265  240  303  39.20  1.79  0.76  0.88  1.04  1.24  **8.12**  2.49  2.96  1.74  0.92  2,479  2,333  1,312  2,953  3,889  1,648  1,952  1,610  1,610  2,413  1,561  2,236  1,409  1,903  10,829  6,638  4,255  26,192  5,976  28,445  68,082  10,367  4,188  2,127  723  471  637  33.18  39.14  0.55  0.65  0.83  1.05 | HFrEF  **11.90**  6.44  3.66  1.81  -  4,174  4,579  2,526  4,394  2,520  2,779  2,675  2,804  4,969  3,257  4,826  4,560  3,199  15,289  6,166  2,981  8,557  8,581  73,983  7,294  18,303  7,426  1,611  682  520  629  18.60  9  -  -  -  -  **9.19**  4.17  3.18  1.84  -  2 951  4 140  1 944  3 855  2 445  2 398  1 876  4 615  3 929  1 770  3 288  4 560  1 517  11 363  3 209  2 736  23 299  6 575  44 864  6 903  13 906  6 221  871  265  240  303  39.20  1.79  -  -  -  -  **5.66**  2.15  1.87  1.65  -  2,479  2,333  1,312  2,953  3,889  1,648  1,952  1,610  1,610  2,413  1,561  2,236  1,409  1,903  10,829  6,638  4,255  26,192  5,976  28,445  68,082  10,367  4,188  2,127  723  471  637  33.18  39.14-  -  -  -  - | HFpEF  **9.09**  4.95  2.55  1.60  -  4,174  4,579  2,526  4,394  2,520  2,779  2,675  2,804  4,969  3,257  4,826  4,560  3,199  15,289  6,166  2,981  8,557  8,581  73,983  7,294  18,303  7,426  1,611  682  520  629  18.60  9  -  -  -  -  **6.66**  3.06  1.89  1.71  -  2 951  4 140  1 944  3 855  2 445  2 398  1 876  4 615  3 929  1 770  3 288  4 560  1 517  11 363  3 209  2 736  23 299  6 575  44 864  6 903  13 906  6 221  871  265  240  303  39.20  1.79  -  -  -  -  **8.02**  2.95  3.31  1.76  -  2,479  2,333  1,312  2,953  3,889  1,648  1,952  1,610  1,610  2,413  1,561  2,236  1,409  1,903  10,829  6,638  4,255  26,192  5,976  28,445  68,082  10,367  4,188  2,127  723  471  637  33.18  39.14  -  -  -  - | **€ per patient per day**  € per patient per day  € per patient per day  € per patient per day  € per patient per day  € per unit  € per unit  € per unit  € per unit  € per unit  € per unit  € per unit  € per unit  € per unit  € per unit  € per unit  € per unit  € per unit  € per unit  € per unit  € per unit  € per unit  € per unit  € per unit  € per unit  € per unit  € per unit  € per bed-day  € per bed-day  € per bed-day  € per bed-day  € per bed-day  € per bed-day  € per bed-day  € per bed-day  € per bed-day  € per bed-day  **€ per patient per day**  € per patient per day  € per patient per day  € per patient per day  € per patient per day  € per unit  € per unit  € per unit  € per unit  € per unit  € per unit  € per unit  € per unit  € per unit  € per unit  € per unit  € per unit  € per unit  € per unit  € per unit  € per unit  € per unit  € per unit  € per unit  € per unit  € per unit  € per unit  € per bed-day  € per bed-day  € per bed-day  € per bed-day  € per bed-day  € per bed-day  € per bed-day  € per bed-day  € per bed-day  € per bed-day  **€ per patient per day**  € per patient per day  € per patient per day  € per patient per day  € per patient per day  € per unit  € per unit  € per unit  € per unit  € per unit  € per unit  € per unit  € per unit  € per unit  € per unit  € per unit  € per unit  € per unit  € per unit  € per unit  € per unit  € per unit  € per unit  € per unit  € per unit  € per unit  € per unit  € per unit  € per unit  € per bed-day  € per bed-day  € per bed-day  € per bed-day  € per bed-day  € per bed-day  € per bed-day  € per bed-day  € per bed-day  € per bed-day | NA |  |
| Scalvini S et al. [33] | NA | 1 year | Home-based telecardiology vs UC | Patients  **Total costs**   Hospitalization charges   Telecare service charges | 230  **107,494**  95,688  11,806 | | 230  **140,874**  140,874 | | Number  €  €  € | NA |  |
| Ekman M et al. [37] | 1999 | 1.3 years | Bisoprolol vs Placebo | **Total costs**  Hospitalization  Bisoprolol  Other medication  Dosage titration  Added life-years  Reason for admission   HF worsening   Total (HF + any disease)  Ward   Cardiology   ICU   General   Other | **109,791**  2,053  177  846  723  105,991  472 (2,967)  2,053  446  597  374  569 | | **103,655**  2,484  -  840  -  100,331  883 (2,967)  2,484  446  597  374  569 | | **€ per patient**  € per patient  € per patient  € per patient  € per patient  € per patient  € per patient (€ per admission)  € per patient  € per day  € per day  € per day  € per day | NA |  |
| Gonzalez-Loyola FE et al. [41] | 2010-2013 | 4 years | After diagnosis vs Before diagnosis | **Total costs**  Nurse at PHC  Nurse at patient's home  Laboratory test  GP at PHC  GP at patient's home  PC emergency visits | **113,084,893 (887.50)**  34,234,788 (268.68)  14,339,790 (112.54)  1,500,235 (11.77)  52,835,560 (414.67)  8,336,120 (65.42)  1,838,400 (14.42) | | **110,541,278**  **(664.19)**  33,551,756 (201.60)  8,182,170 (49.16)  1,642,832 (9.87)  61,054,560 (366.85)  5,605,600 (33.68)  504,360 (3.03) | | € (€ per patient per year)  € (€ per patient per year)  € (€ per patient per year)  € (€ per patient per year)  € (€ per patient per year)  € (€ per patient per year)  € (€ per patient per year) | NA |  |
| Grustam AS et al. [47] | 2015 | 20-year horizon | HTM and NTS vs UC | **Total cost**  Telemonitoring costs   Equipment and service fee   Installation fee  Personnel costs  Healthcare   Nurse   GP   Specialist   Hospitalist   Telephone call   Telehealth nurse  Non–healthcare   Telenurse overhead  Hospital-related costs   Day in a hospital   ER visit | HTM  **27,186**  800-1,500  50-150  20  28  64  64  14  30,000  10,000  435  151 | NTS  **24,604**  800-1,500  50-150  20  28  64  64  14  30,000  10,000  435  151 | UC  **14,414**  -  -  20  28  64  64  -  -  -  435  151 | | **€ per patient**  € per year  € every 5 years  € per visit  € per visit  € per visit  € per visit  € per televisit  € per year  € per year  € per patient per day  € per patient per visit | 4% |  |
| González-Guerrero JL et al. [48] | 2010 | 1 year | DMP vs UC | **Total sanitary system costs**  **Total societal costs**  Health-care costs   Hospitalization   Specialist office visits    New patient    Follow-up   Telephone contacts    Physician    Nurse   PC practice visits    Office    Home   Emergency visits   Rehabilitation   Medications  Non-health costs   Transportation   Caregiver time   Residential care   Cost of the intervention | **6,458.99**  **7,514.79**  4,732.04  20.52  309.52  89.69  5.90  955.64  74.94  122.17  5.40  143.17  107.15  270.26  678.39  728.13 | | **8,079.76**  **8,589.43**  6,449.85  23.85  69.77  -  -  1,119.40  127.05  170.44  -  119.40  -  -  509.67  - | | € per patient  € per patient  € per patient  € per patient  € per patient  € per patient  € per patient  € per patient  € per patient  € per patient  € per patient  € per patient  € per patient  € per patient  € per patient  € per patient | NA |  |
| Comín-Colet J et al. [50] | 2010-2013 | 6 months | Telemedicine+ UC vs UC | **Total healthcare costs** | **4,400** | | **8,000** | | **€ per patient** | NA |  |
| Sahlen KG et al. [51] | 2011-2013 | 6 months | PREFER vs UC | Patients  **Total cost**  GP  Other medical professionals  Emergency transport  Hospital care  **Total cost**  GP  Other medical professionals  Emergency transport  Hospital care  **Average cost**  **Max-min cost**   Ambulance transport   Physician   RN, physiotherapist, and occupational therapist   Hospital care   Travel expenses | 36  **146,889**  16,468  68,103  3,525  58,793  **4,078**  457  1,890  98  1,632  **756**  **5,643-141**  320  5,990  3,060  570  0.2 | | 36  **206,301**  8,075  6,807  15,061  176,357  **5,727**  224  189  418  4,896  **1,061**  **5,069-15**  320  5,990  3,060  570  0.2 | | Number  **€**  €  €  €  €  € per patient  € per patient  € per patient  € per patient  € per patient  **€ per patient per month**  **€ per patient per month**  € per transport  € per month  € per month  € per day  € per km | NA |  |
| Postmus D et al. [57] | 2009 | 18 months | BS and IS vs UC | **Total cost**  Intervention   Inpatient hospital visits   Home visits   Outpatient visits   Telephone contacts   Multidisciplinary advice  Hospitalization   CV related   Non-CV related   Short-stay admission   HF-related diagnostics  Short-stay admission  HF-related diagnostics | BS  **9,616**  707  29  59  110  5  31  8,125  769  522  251  Various  45  1,052 | IS  **10,794**  1,055  29  59  110  5  31  8,818  769  522  251  Various  43  1,249 | UC  **9,693**  376  29  59  110  5  31  8,458  769  522  251  Various  41  1,065 | | **€ per patient**  € per patient  € per visit  € per visit  € per visit  € per call  € per visit  € per patient  € per day  € per day  € per admission  € per procedure  € per patient  € per patient | NA |  |
| De la Porte PW et al. [58] | 2000-2003 | 12 months | Intensive follow-up vs UC | Patients  **Total costs**  Hospitalization  HF clinic program (including a nurse, a physician, a dietician, a lab and ECGs) | 118  **115,292**  65,046  50,246 | | 122  **202,728**  202,728  - | | Number  **€**  €  € | NA |  |
| del Sindaco D et al. [59] | 2001-2002 | 2 years | DMP vs UC | Patients  **Total costs**  HF admission  Nurse  Cardiologists | 86  **163,916.22**  149,660.22  6,352.00  7,904.00 | | 87  **248,372.28**  248,372.28  -  - | | Number  €  €  €  € | NA |  |
| Agvall B et al. [60] | 1999-2000 | 2 years | NA | **Total cost**  Hospital care   Inpatient care    Stay/day in an ICU    Stay/day in a hospital ward   Outpatient care    Visit to a physician    Visit to a HF nurse  PHC   Cost related GP    Visit    Home visit    Telephone contact    Prescription of drugs   Cost related to nurses    Visit (regular nurse)    Visit to an asthma nurse    Visit to a diabetic nurse    Visit to a hypertension nurse   Cost related to district nurse    Visit    Home visit   Cost related paramedical staff    Occupational therapist    Physiotherapist    Chiropodist   Costs related to other resources    Stay/day in a nursing home    Nursing home    Cost for medication    Cost for chest X-ray    Physiology examinations | **4,626**  155  1,771  215  23  561  12.98  1.75  3  7.61  10.98  51.8  14.98  86.88  194.10  50.80  19.22  25.59  52.05  229.05  875.89  288.47  27.84 | | NA | | **€ per patient per year**  € per patient per year  € per patient per year  € per patient per year  € per patient per year  € per patient per year  € per patient per year  € per patient per year  € per patient per year  € per patient per year  € per patient per year  € per patient per year  € per patient per year  € per patient per year  € per patient per year  € per patient per year  € per patient per year  € per patient per year  € per unit  € per patient per year  € per patient per year  € per patient per year  € per patient per year | NA |  |
| Atienza F et al. [61] | 1999-2000 | 509 days (median) | Close follow-up vs UC | **Total cost**  Hospital readmissions   Hospitalization  Intervention   Cardiologist   Nursing   Telephone calls | **3,354**  2,912  451  442  29,789  40  10 | | **5,417**  5,417  451  -  -  -  - | | **€ per patient**  € per patient  € per day  € per patient  € per year  € per hour  € per call | NA |  |
| Ledwidge M et al. [62] | 1998-2000 | 3 months | MC vs RC | **Total cost**  Hospitalization  Intervention   Salaries (nurses and dieticians)    Nursing    Diet   Unscheduled contacts | **9,974**  4,114  5,860  31,750  3,680  1,938  241 | | **47,190**  47,190  -  -  -  -  - | | **€**  €  €  € per specialist per year  €  €  € per visit | NA |  |

ADHF: Acute Decompensated Heart Failure; BS: Basic Support; CABG: Coronary Artery Bypass Graft; CCU: Coronary Care Unit; CKD: Chronic Kidney Diasease; CV: Cardiovascular; CVD: Cardiovascular Disease; DMP: Disease Management Program; ECG: Electrocardiogram; ER: Emergency Room; GP: General Practitioner; HF: Heart Failure; HFpEF: Heart Failure Preserved Ejection Fraction; HFrEF: Heart Failure Reduced Ejection Fraction; HD: High Dose; ICU: Intensive Care Unit; IS: Intensive Support; LD: Low Dose; MC: Multidisciplinary Care; MI: Myocardial Infarction; MRI: Magnetic Resonance Image; NA: Not Applicable; PAD: Peripheral Artery Disease; PC: Primary Care; PHC: Primary Healthcare; PTCA: Percutaneous Transluminal Coronary Angioplasty; RC: Routine Care; RN: Registered Nurse; ST: Standard Therapy; HTM: Home Telemonitoring; NTS: Nurse Telephone Support; UC: Usual Care; UK: United Kingdom

**Supplementary Table S4:** Heart failure costs for usual care in some European countries

| **Country** | **Year of valuation (range)** | **Costs per patient per year (min-max in €)** | **References** |
| --- | --- | --- | --- |
| Spain | 1999-2019 | 888-19,642 | [16,17,25,41,48,50,61] |
| Germany | 2003-2019 | 2,986-22,647 | [16,28,32] |
| UK | 2003-2019 | 2,551-15,550 | [16,32] |
| Denmark | 1998-2016 | 17,093 | [18] |
| Greece | 2009-2011 | 16,908 | [20] |
| Ireland | 1998-2013 | 4,016-13,011 | [21,62] |
| Italy | 2001-2011 | 613-6,283 | [24,33,59] |
| Poland | 2011 | 1,794 | [26] |
| Belgium | 2001 | 4,852 | [29] |
| France | 2003 | 3,942 | [32] |
| Sweden | 1999-2013 | 2,557-11,461 | [37,51,60] |
| The Netherlands | 2000-2015 | 1,662-6,462 | [47,57,58] |

UK: United Kingdom

**Supplementary Table S5:** Cost-effectiveness analyses regarding heart failure disease management programs and drugs in clinical assays

| **Reference** | **Valuation year** | **Follow-up** | **Intervention vs Comparator** | **Unit** | **Difference (Intervention minus Comparator)** | | | |
| --- | --- | --- | --- | --- | --- | --- | --- | --- |
| McEwan P et al. [16] | 2019 | 18.2 months (median) | Dapagliflozin  added to ST vs ST | **UK**  LY  QALY  ICER (€/QALY)  **Germany**  LY  QALY  ICER (€/QALY)  **Spain**  LY  QALY  ICER (€/QALY) | 0.58  0.48  6,828  0.61  0.5  5,379  0.61  0.5  9,406 | | | |
| McMurray JJ et al. [32] | 2003 | 41 months | Candesartan added to conventional treatment vs Placebo i.e. conventional treatment for HF | LY  **France**  Cost/LY (€)  **Germany**  Cost/LY (€)  **Spain**  Cost/LY (€) | 0.068  Dominant (cost-saving)  2,997  1,348 | | | |
| Ekman M et al. [37] | 1999 | 1.3 years | Bisoprolol vs Placebo | LY  ICER (€/LY) | 0.291  19,216 (including costs of added LY)  1,490 (excluding costs of added LY) | | | |
| Grustam AS et al. [47] | 2015 | 20-year horizon | HTM and NTS vs UC | LY  QALY  ICER (€/LY)  ICER (€/QALY) | HTM vs NTS  −0.19  −0.14  −16,555  −23,611 | NTS vs UC  1.51  1.16  6,363  8,270 | | HTM vs. UC  1.31  1.02  9,726  12,479 |
| González-Guerrero JL et al. [48] | 2010 | 1 year | DMP vs UC | QALY  ICER (€/QALY) | Sanitary system  0.0423  −38,274 | | Societal  0.0423  −25,390 | |
| Sahlen KG et al. [51] | 2011-2013 | 6 months | PREFER vs UC | QALY  Cost/QALY (€) | 0.25  −14,853 | | | |

DMP: Disease Management Program; HF: Heart Failure; HTM: Home Telemonitoring; ICER: Incremental Cost-Effectiveness Ratio; LY: Life Year; NTS: Nurse Telephone Support; QALY: Quality-Adjusted Life Year; ST: Standard Therapy; UC: Usual Care; UK: United Kingdom

**Figures**

**Supplementary Figure 1**: Bar graphs comparing annual HF-related costs per patient under UC versus DMP. UC denotes standardized clinical management, while DMP includes structured interventions such as home-based telecardiology [33], DMP [48,59], telemedicine [50], PREFER [51], IS [57], intensive follow-up [58,61] and MC [62].

DMP: Disease Management Program; HF: Heart Failure; HFpEF: Heart Failure preserved Ejection Fraction; HFrEF: Heart Failure reduced Ejection Fraction; IS: Intensive Support; MC: Multidisciplinary Care; PREFER: Palliative advanced home caRE and heart FailurE caRe; UC: Usual Care

**Supplementary Figure 2**: Bar graph comparing average annual HF-related costs per patient between those with HFrEF and HFpEF [21]

GP: General Practitioner; HF: Heart Failure; HFpEF: Heart Failure preserved Ejection Fraction; HFrEF: Heart Failure reduced Ejection Fraction
