# Supplementary figures and images for: Economic burden of heart failure in Europe: A systematic review of costs and cost‐effectiveness
Source: ESC Heart Fail. 2025 Nov 26;12(6):4055–68. doi: 10.1002/ehf2.70017 (PMC12719840; doi:10.1002/ehf2.70017)

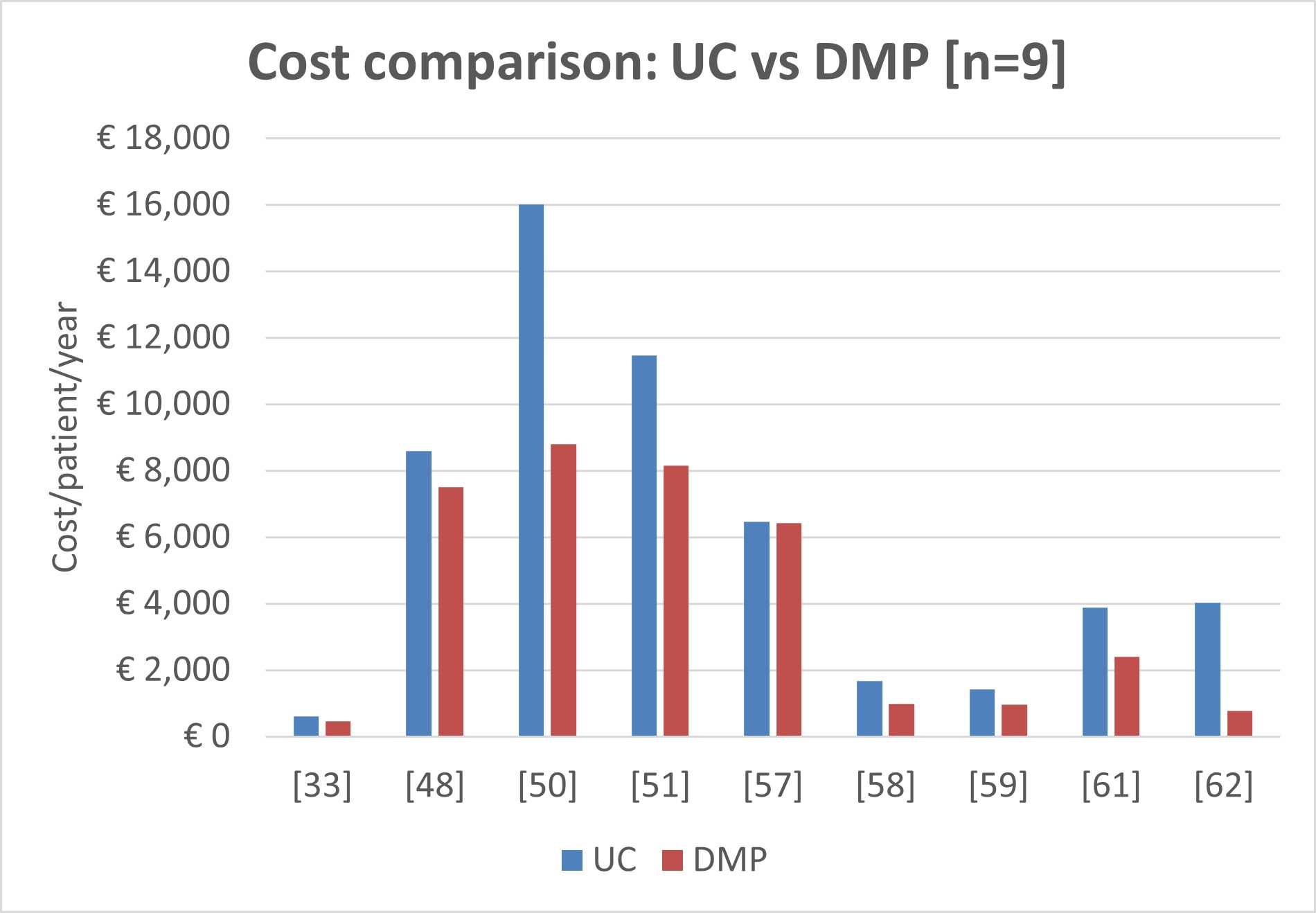

Supplement: Supplementary file 2 — Figure S1. Bar graphs comparing annual HF‐related costs per patient under UC versus DMP. UC denotes standardized clinical management, while DMP includes structured interventions such as home‐based telecardiology [33], DMP [48,59], telemedicine [50], PREFER [51], IS [57], intensive follow‐up [58,61] and MC [62]. [file EHF2-12-4055-s001.jpg]

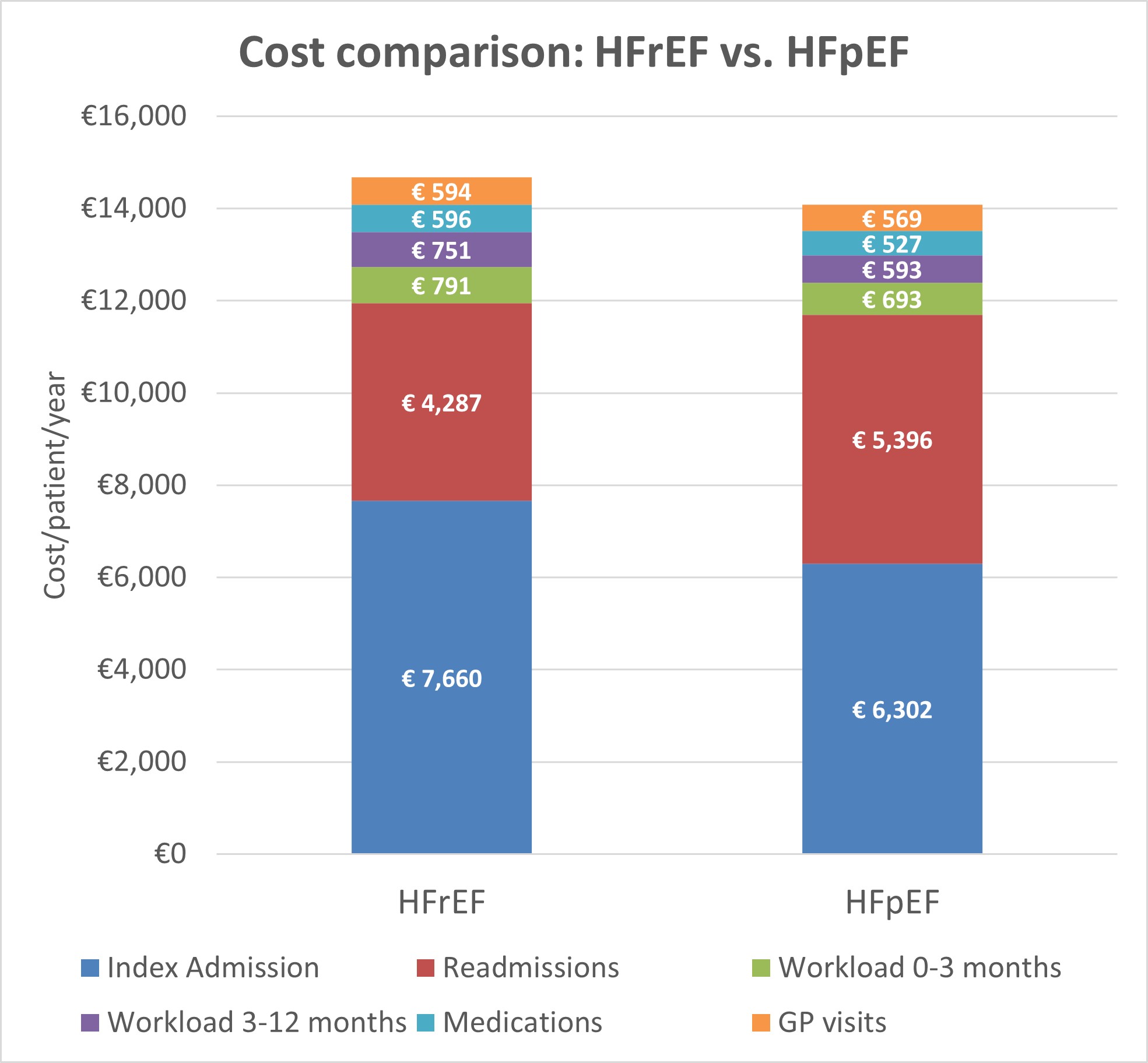

Supplement: Supplementary file 3 — Figure S2. Bar graph comparing average annual HF‐related costs per patient between those with HFrEF and HFpEF [21]. [file EHF2-12-4055-s002.jpg]
